# The potential for cascading failures in the international trade network

**DOI:** 10.1371/journal.pone.0299833

**Published:** 2024-03-01

**Authors:** Heesuk Kang, Kyu-Min Lee, Jae-Suk Yang

**Affiliations:** 1 Graduate School of Future Strategy, Korea Advanced Institute of Science and Technology, Daejeon, Republic of Korea; 2 KT Sat, Seoul, Republic of Korea; 3 College of Business, Korea Advanced Institute of Science and Technology, Seoul, Republic of Korea; Hosei University: Hosei Daigaku, JAPAN

## Abstract

In our study, we introduce indicators that quantify the influence of each country in complex trade scenarios involving the exchange of raw materials, intermediate goods, and final products across multiple countries. We systematically employ an agent-based model to simulate the propagation of failures from one node to the entire network. This approach allows for the assessment of the impact of each country and the identification of patterns of interaction in the multi-step trade network. Unlike conventional analyses of trade networks, which depict straightforward single-step import/export transactions, our approach captures the intricate realities of processes like raw material procurement, production, and sales in numerous countries from a macroscopic perspective. The findings of our analysis of trade data spanning from 1990 to 2022 reveal several key insights. Firstly, sensitivity to changes in trade volume leading to global failures within interconnected networks has intensified over time. The potential of failure propagation across countries has increased over time, as has the interconnectedness of countries in the global trade landscape. Secondly, despite the increased sensitivity to changes in global trade volume, many countries have become less vulnerable to the influence of others within their multi-step trade networks. This trend aligns with deglobalization, which is evidenced by events such as Brexit and the surge in protectionist measures; these changes indicate a shift in the balance of influence within global trade networks. Thirdly, the results of our analysis of the relationship between load changes and global failures from a regional perspective reveal an intriguing phenomenon: despite limited direct trade connectivity, the interaction between the Latin American and Sub-Saharan African regions is considerable. This suggests the existence of hidden connections between intermediary countries, such that one region’s actions can alter the load sensitivity of another, impacting them in unforeseen ways. Furthermore, intra-regional interactions are diminishing in East Asia, while Europe is experiencing a gradual increase in interactions. These trends reflect evolving regional influence, the dynamics of geographic proximity, and the results of economic integration efforts. Additionally, even though the observed period was not long enough to confirm a long-term trend, the previous trend direction was affirmed to persist despite a temporary decrease in trading and reduced sensitivity due to the COVID-19 pandemic. Our study highlights the complexity of global trade dynamics and the need to consider multi-step trade networks and their potential cascading effects when analyzing trade patterns and vulnerabilities.

## 1. Introduction

Understanding vulnerability in the global trade network is imperative for identifying potential weak points in this complex web of connections. Susceptible to disruptions such as natural disasters, geopolitical events, or economic crises, the global trade network requires a thorough assessment of potential economic impacts to enhance supply chain resilience and prevent disruptions in one part of the chain from triggering widespread economic or industrial downturns. Examination of cascading effects within global trade networks is particularly important given historical instances where crises originating in one country swiftly permeated neighboring countries, at times having global consequences [1, 2]. Milestones such as the Asian financial crisis of 1997–1998 and the global economic downturn in 2008 underscored the interconnectedness of nations through financial flows and trade routes and the global repercussions of such crises [3–7].

The cascading effect in global trade networks has been studied extensively based on cross-border trade data [8–11]. When a failure occurs at one node in the network, it does not remain confined within that nation’s borders, but extends to neighboring nodes. When failures accumulate, subsequent failures in adjacent nodes are triggered, eventually leading to the collapse of the entire system [12]. Data regarding direct transactions provides the most intuitive insights into the connection between products and geographic markets. However, the trade network, once viewed through the lens of bilateral transactions, has transformed into a complex and intricate web of interconnected processes spanning multiple countries and characterized by non-linear value chains. In fact, because multiple countries engage in the exchange of raw materials, intermediate goods, and final products, a global supply chain has emerged [13]. In contemporary trade, interaction among these countries involves multiple steps; therefore, relying solely on single-step export and import figures is inadequate for capturing the impact of intermediate countries in this chain.

To address this complexity, researchers have turned to advanced tools like the World Input-Output Database (WIOD) [14], which is segmented by sectoral level for each country. This approach provides a more nuanced understanding of the interdependencies within the global production network. However, quantifying trade data from countries worldwide into an IOD through a bottom-up approach presents significant challenges. The sheer complexity and scale of the global economy, coupled with variations in data availability and quality across countries, create formidable tasks for researchers. Consequently, there is a need to move away from reductionist analyses that rely on incomplete national-level sectoral data. Instead, researchers should adopt a comprehensive approach that considers each country’s role within this multi-step trade paradigm. This shift in methodology provides a more holistic understanding of the dynamics at play in the global trade landscape.

In response to these challenges, agent-based models can be invaluable for simulating complex systems and assessing the potential impacts of failures within multi-step trade networks. In this study, we simulate the propagation of failures from one node in the network to the entire system. We identify indicators that measure the extent to which failures originating in one country propagate globally rather than being limited to neighboring nations and assess the position of each country in the multi-step trade network by examining indicators over time. This approach allows us to quantify the potential for cascading failures in each country and the influence of each country on others. Avoiding the division of data by industry, this study pioneers a novel method that provides a nuanced and comprehensive understanding of the dynamics of cascading failures in global trade networks.

From the 1990s to the 2020s, numerous pivotal political and economic events took place, and globalization peaked. Key milestones occurred, such as the reunification of Germany, the dismantling of the former Soviet Union, China’s accession to the WTO, and the establishment of the European Union, all of which affected global trade connectivity. In the post-global financial crisis of the late 2000s, protectionism increased, and the ongoing impact of COVID-19 also slowed the process of globalization.

By setting our research period from 1990 to 2022, we capture changes in trade connectivity and quantify potential chain effects within the multi-step trade network. Moreover, we confirm the impact of COVID-19, which other studies have not attempted due to data limitations. Through a holistic lens, we provide a macroscopic perspective while analyzing shifts in trade patterns and vulnerabilities in the global trade network.

## 2. Model

In our analysis, we employ an agent-based model. Fluctuations in trade volume caused by a policy change in a particular country, such as the implementation of a protective tariff, can cause failures in that country that can spread globally depending on the country’s position in the trade network. The cascading failure model utilized herein has been studied by many scholars [15–19]. Due to integration in the global economy, shocks or failures that originate in one country may be transmitted to its economic partners through various channels, which may explain numerous economic phenomena. We utilize a parameter that indicates when a failure in one country begins to spread globally as an index to evaluate that country’s influence within a globally interconnected trade network.

### 2.1 Data

To create this model, we meticulously compiled comprehensive data encompassing the Gross Domestic Product (GDP) of over 200 countries around the world (measured in current USD) and the corresponding export and import activities between them (also measured in current USD). This extensive dataset spans a timeframe of 33 years, specifically from 1990 to 2022. Data for essential economic indicators can be sourced from a variety of reputable institutions, including the World Bank, International Monetary Fund (IMF), World Trade Organization (WTO), UN Comtrade, and UNCTAD. To ensure data consistency, this study specifically employed GDP figures from the World Bank and export/import transaction volume data from the IMF. This approach was taken to guarantee an equivalent data collection process for both GDP and trade volumes to reflect their conversion to current USD accurately throughout the study period (1990–2022).

The World Trade Network is depicted as a weighted directional network, wherein nodes symbolize countries according to their respective GDPs, while edges reflect their magnitudes of import and export flows. Pertaining to trade volume, the IMF furnishes data regarding both CIF (Cost, Insurance, and Freight) and FOB (Free on Board). For this investigation, CIF data was selected. In theory, the value of exports from country A to country B should match the value of imports from country B to country A. However, in practice, exporting countries frequently furnish FOB data, whereas importing nations provide CIF data for statistical purposes. Recognizing that import data tends to be more accurate compared to export data due to reporting inconsistencies, we opted for CIF data to ensure uniformity and reliability in the results of the analysis.

We classified the regions into seven categories according to the classification system provided by the World Bank: East Asia & Pacific, South Asia, Middle East & North Africa, Europe & Central Asia, Sub-Saharan Africa, North America, and Latin America & Caribbean.

To ensure consistency, the most recent names were used for countries whose names had been changed. Data from the former Soviet Union and Russia until 1999 were mixed; therefore, discussion of the economic effects of changes in Russia in the 1990s seemed difficult. Despite the potential inaccuracies in data from various countries, trends and scales similar to those provided by global macro indicators are evident in the network analysis. Therefore, we deem it to be reasonable to present insights on cascading failures from a macroscopic perspective in this study. All the data that were used in this study are included in S1 and S2 Data.

### 2.2 Cascading failure process

We simulate cascading failures in a trade network when disruptions or failures, such as natural disasters, economic crises, or supply chain disruptions, occur [17]. When a failure transpires within a node, the load is transferred to its connected nodes. If the accumulated load in a connected node surpasses its capacity, a second failure may also occur in that node. This process may continue, with the load being transferred to other connected nodes, after which third, fourth, and subsequent failures may occur. The simulation continues until there are no additional nodes experiencing failures. Unlike the model presented in a previous study [17], in which the total number of failure nodes was counted and the transmitted load rate remained constant, this study examines the load rate at the point when failures are transmitted to all nodes in the network. This is considered the point at which failure originating in a specific country is transmitted to the global trade network. This new indicator captures the influence on the trade network of the country in which the initial failure occurred.

#### 2.2.1 Node capacity

In this study, the capacity of node *i*, *C*_*i*_, to resist failure is defined as an amount proportional to the node country’s GDP, as follows:

$$C_{i}=t\times GDP_{i},i=1,2,\ldots ,n,$$

where *t* is a parameter representing the capacity in the range of 0<*t*<1.

#### 2.2.2 Redistribution of load and cascading failure: Evaluation index

When a failure first occurs at node *i*, the load as much as the *f* ratio of the weight of the link connected to *i* is transferred to all nodes *j* connected to node *i*. The cumulative load delivered to node *j*, *L*_*j*_, is defined as follows:

$$L_{j}=\max\left\{\sum_{k}\Delta W_{jk},\sum_{k}{\Delta W}_{kj}\right\}=max\{\sum_{k}f\times \Delta Export_{jk},\sum_{k}f\times \Delta Import_{kj}\}$$

If the cumulative load delivered to node *j* exceeds the capacity of node *j* (*L*_*j*_>*C*_*j*_), node *j* also fails.

This load redistribution process is repeated until there are no more failed nodes.

In our model, failure occurs in one country for some reason, and that failure undergoes several intermediate stages in a complex trade network until it reaches the last connected country. At the last stage, if the index of the load that propagates the failure to the entire network is evaluated, the influence of the country in the multi-step trade network can be quantified and visualized.

The number of nodes in which cascading failure occurs due to the initial failure after which no further failure occurs is called avalanche size, the value for which is obtained by determining the number of failure nodes when $\frac{max\{\sum_{k}\Delta Export_{jk},\sum_{k}\Delta Import_{kj}\}}{C_{j}}>(t/f)$. Thus, avalanche size is determined using the combined variable (*t*/*f*), which is not affected by individual *t* and *f*. We first look at how the avalanche size changes according to changes in the value of (*t*/*f*). If avalanche size according to changes in the value of (*t*/*f*) is determined by changing the *f* value representing the failure load and the capacity parameter *t* remains constant, a discontinuity point such as a first-order phase transition exists. Before and after this point, avalanche size shows a local level of cascading failure, after which a rapid discontinuity occurs, resulting in a cascading failure on a global level, where failure occurs in most countries. The value of (*t*/*f*) at which the normalized avalanche size approaches 1 while passing through the point of discontinuity is used as the evaluation index. (*t*/*f*) at this critical point is denoted as ${\left(t/f\right)}_{c_{i}}$, defined as the Cascading Failure Critical Point (CFCP) of country *i*. CFCP is a function of the reciprocal of the rate of change in load, *f*, which is determined by the amount of trade with country *i*’s trade partners, and their GDPs. A larger CFCP means that a failure can be transmitted globally at a lower load fluctuation rate. This can be interpreted as follows: country *i* is very influential in the implicit multi-step trade network.

The main objective of this simulation is to identify and compare the threshold value (*t*/*f*)_*c*_ at which most countries in the network experience collapse. When the failure load value, represented by *f*, is relatively low, and the ratio of (*t*/*f*) is greater than that of (*t*/*f*)_*c*_, the failure remains localized, affecting only the epicenter country. The detailed process can be found in S1 Appendix.

As the failure load *f* increases and the ratio of (*t*/*f*) becomes less than that of (*t*/*f*)_*c*_, the localized failure transforms into a global cascade, with failure spreading to most countries across the entire network. At a critical point (*t*/*f*)_*c*_, the avalanche size exhibits a discontinuous increase, resembling a staircase pattern. However, in some countries, the avalanche size is represented by multiple staircases instead of one. In such cases, it is necessary to establish a reference to identify the critical point.

The CFCP of country *i*, (*t*/*f*)_*c*_ is defined as the critical value observed when more than 60% of the countries in the network experience collapse after a failure originating in country *i*. Even if the reference point is set at a different value, that is, 70% or 50% of the countries collapse instead of 60%, the correlation between the results under each criterion remains consistent (correlation value = 1). Therefore, we conclude that the threshold value (*t*/*f*)_*c*_ is not sensitive to the specific criterion used to determine the percentage of countries experiencing collapse.

For this study, the value of avalanche size exceeding 60% is adopted as (*t*/*f*)_*c*_, as it provides a robust and reliable measure to assess the propagation of failures within the network.

**Fig 1:**
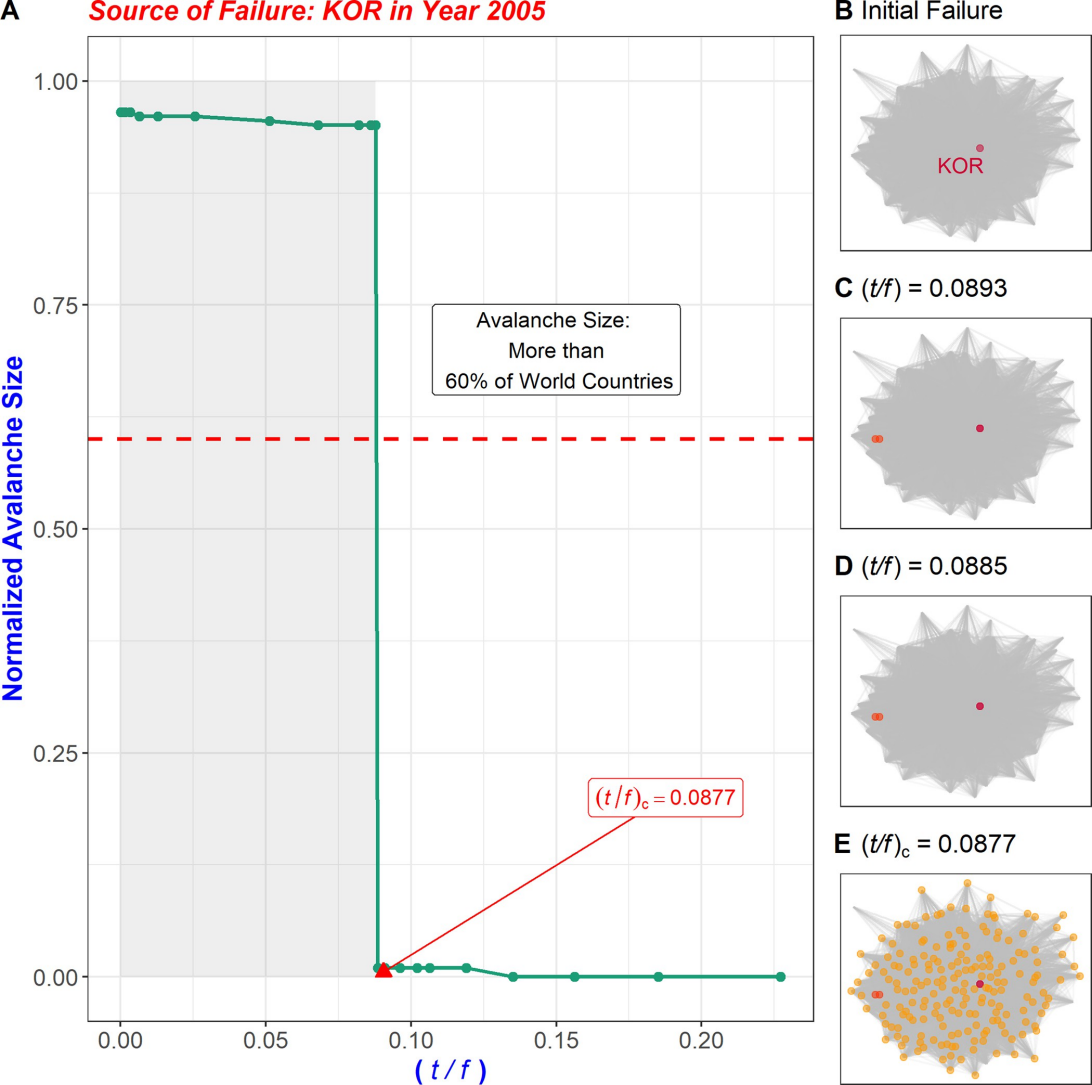
Normalized avalanche size with respect to changes in (*t*/*f*) value. (A) In this simulation, a failure originating in an epicenter country is propagated to neighboring countries. As the load *f* increases and the value of (*t*/*f*) gradually decreases (moving from the right to the left of the x-axis), the graph displays the change in the number of countries to which the failure is transmitted. The avalanche size represents the number of countries to which the failure has been delivered, indicating the critical point defined as CFCP (Cascading Failure Critical Point). At this point, the failure spreads instantaneously to all countries in the network rather than increasing linearly based on the change in (*t*/*f*) value. The graph depicts the failure nodes at different stages: (B) the initial failure stage, (C) with (*t*/*f*) = 0.0893, (D) with (*t*/*f*) = 0.0885, and (E) with (*t*/*f*) = 0.0877. In the trade network, following the occurrence of a failure in the first country, as the (*t*/*f*) value decreases, the countries to which the failure is transmitted are displayed. (E) The red node indicates the country where the initial failure occurred. As the (*t*/*f*) value gradually decreases from 1, there is not much difference in the failure-delivered countries between (C) and (D). However, the number of countries (yellow) that experience sudden failure increases from (D) to (E). A red node indicates the country in which the failure originated, orange nodes indicate countries to which the failure was transmitted at an intermediate stage, and yellow nodes indicate countries to which the failure was transmitted at the critical point.

Fig 1 shows changes in avalanche size when the failure load *f* spreading to the surrounding nodes changes when an initial failure occurs in KOR (South Korea). Using this methodology, the load that a failure in a given country transmits to the entire system is obtained and quantified as the CFCP, that is, (*t*/*f*)_*c*_.

Using trade network data from 1990 to 2022, and confirming fluctuations in each country’s CFCP value over the 30 years from 1990 to 2022, we can evaluate the potential for cascading failure and the evolution in the multi-step trade network.

The CFCP value defined here considers the load change at the moment when a failure in one country spreads throughout the network and all countries suddenly collapse. A failure originating in one country may be transmitted not only to directly connected neighboring countries, but also to indirectly connected countries through neighboring countries. Multiple steps, that is, secondary and tertiary failures, may occur. This is more than a one-step relationship reflected by exports and imports; it is a multiple-step relationship in which several countries are connected sequentially.

This is comparable to the results of analyses of the Global Value Chain (GVC), where production and consumption take place in various countries, from raw materials to intermediate goods and finished products. GVC analyses employ a bottom-up approach, dissecting data by country and industry and subsequently reclassifying results based on added value. The process of obtaining the CFCP value for each country involves determining the influence of a given country on the most peripheral countries in a network. We herein calculate CFCP by country and check the interdependence between countries in the network from a macroscopic perspective. By identifying nodes that play an important role in the trade network and those that are potentially more vulnerable to failure, we can help policymakers and analysts to develop strategies to mitigate potential risks, enhance resilience, and ensure stability in the event of a failure within the network.

### 2.3 Interconnection between countries

After the node and the links connected to it are deleted and the CFCPs of each remaining node in the network are recalculated, the new CFCP values differ from those of the original network. Through examining this difference, the role of the deleted node in the network can be evaluated, and changes in the role of the node over time can be confirmed. Thus, it is possible to calculate the nonlinear impact of a given node in the network on other nodes directly or indirectly connected to it.

Let us consider the scenario in which country C is removed from the trade network, and failure initially occurs in country A. As a result, all imports and exports involving country C to and from the countries linked with it are reduced to zero. If country C had not been part of country A’s multi-step trade network within the overall trade network, its removal would have caused little change to the CFCP value of country A regardless of the presence or absence of country C. If, however, country C was situated along the path of country A’s multi-step trade network, the transmission of the failure to other parts of the world would inevitably be heightened. This is because reducing the number of paths for transmission of the failure by the removal of country C leads to a more significant load compared to that of the original network configuration. The CFCP value for country A within the modified network without country C is, therefore, reduced.

**Fig 2:**
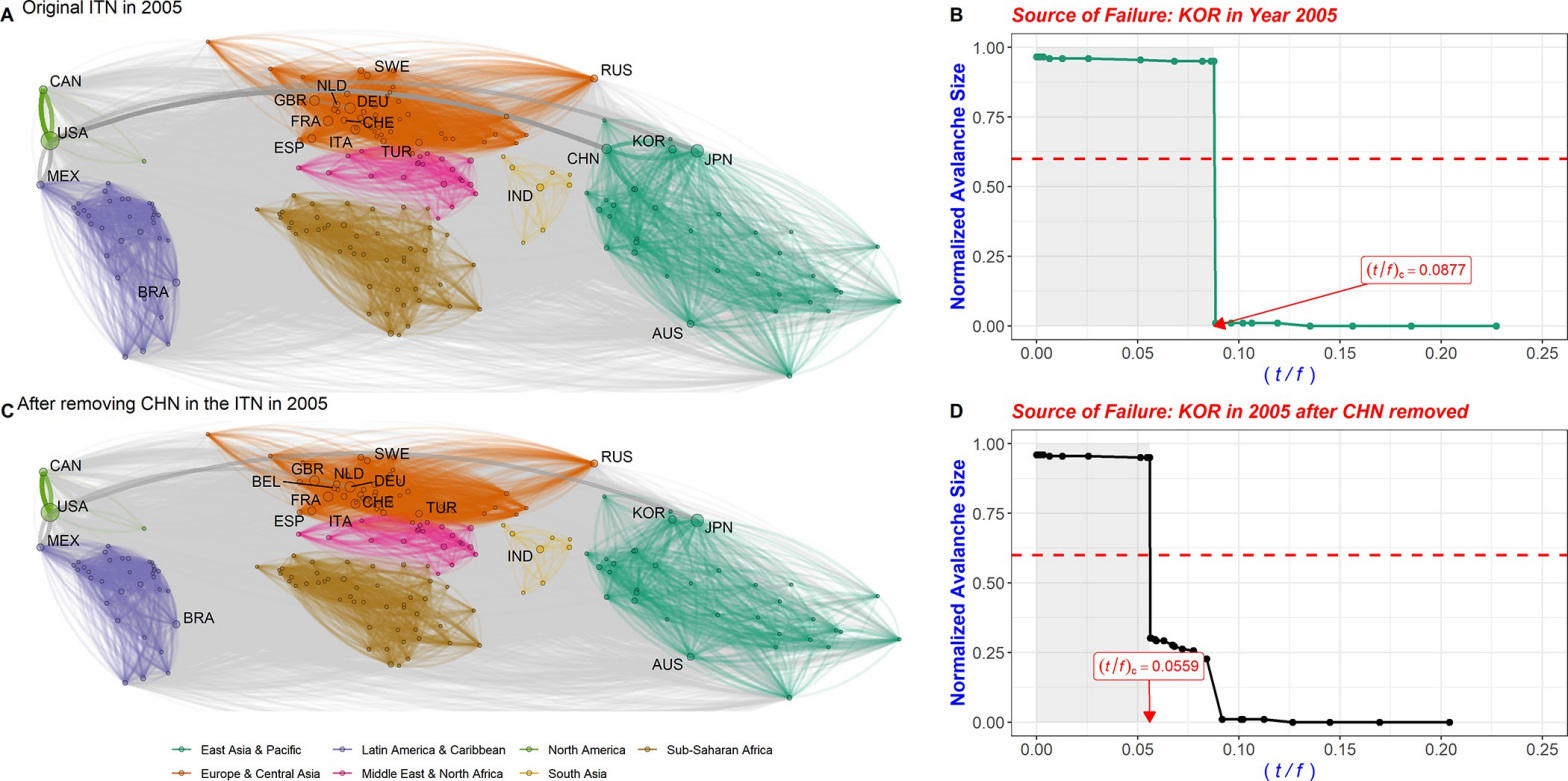
Changes in CFCP between the original trade network and the network after a specific node is removed. (A) The graph portrays the trade network in 2005. Nodes are represented based on their GDP sizes, and the connections (edges) between them signify the volume of imports and exports. (B) When the initial failure happens in KOR within the 2005 trade network, the graph demonstrates a normalized avalanche size and the CFCP value concerning changes in the (*t*/*f*) value. (C) The graph visualizes the trade network after CHN is deleted from it in 2005. (D) The graph shows the changed CFCP value of KOR within the network where CHN has been removed. The difference between the CFCP value of KOR in the original 2005 network and its value in the network with CHN removed indicates that CHN is part of the multi-step trade network involving KOR. This suggests that CHN is influenced by KOR. Edges connecting nodes within the same region are shaded with various colors to indicate intra-regional trade, while edges linking nodes in different regions are gray to represent trade between distinct geographical areas.

Fig 2 shows the change in the CFCP value of the epicenter country after removing one country from the original network. When CHN (China) is deleted from the trade network in 2005, for example, the CFCP value of KOR decreases from 0.0877 to 0.0559 (Fig 2(B) and 2(D)). With CHN outside of KOR’s multi-step trade network, the CFCP value of KOR is unchanged. Since CHN is located inside the multi-step trade network of KOR, however, the CFCP value of KOR does change. From this, we interpret that either KOR influences CHN by a range of values (0.0877–0.0559) in the multi-step trade network or CHN is influenced by KOR by (0.0877–0.0559). Through this process, it is possible to determine the degree of influence in the multi-step trade network between two directly or indirectly connected countries.

#### 2.3.1 Vulnerable value

**Fig 3:**
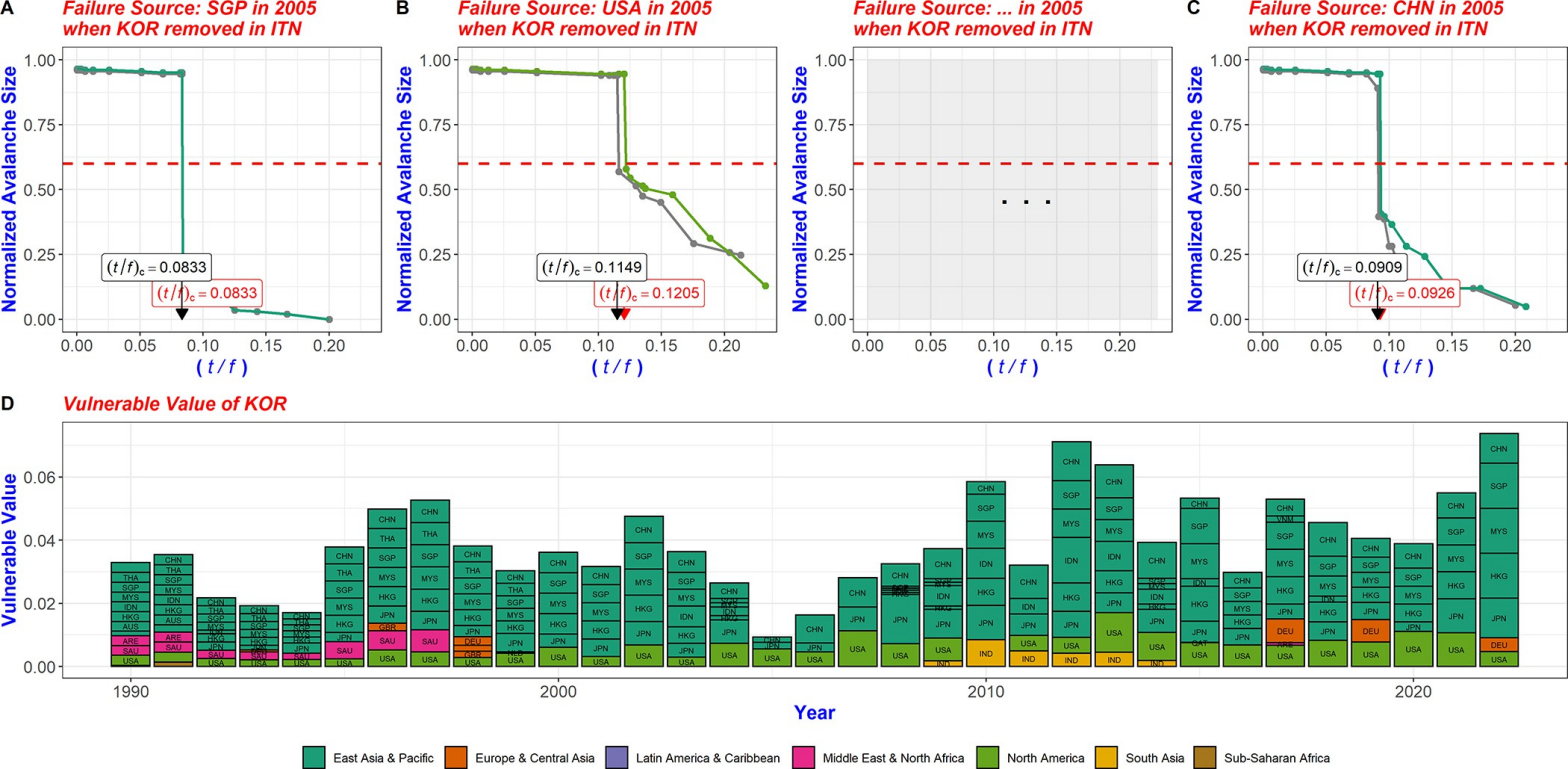
Vulnerable value and evolution of the node through node deletion and CFCP recalculation. (A) There is no change in the CFCP value of the SGP in the network before and after KOR is removed. That is, KOR lies outside SGP’s multi-step trade network, and failure occurring in SGP does not impact KOR across the extensive multi-step trade network. (B) Upon removal of KOR, a disparity in the CFCP values of the USA emerged when comparing the network before and after the deletion. (C) The CFCP value of CHN decreased in the network when KOR was deleted. The insights gained from (B) and (C) indicate that KOR is influenced by both the USA and CHN in the multi-step trade network. (D) The vulnerable value of KOR is computed by analyzing changes in the CFCP values of each country within the network before and after the annual removal of KOR from the trade network, after which these changes are aggregated. Thus, the vulnerable value provides a measure of the influence on KOR of other countries within the multi-step trade network. When KOR was eradicated from the trade network in 2005, the CFCP values of only three countries, namely CHN, JPN, and the USA, underwent changes. This underscores KOR’s vulnerability to these three countries within the multi-step trade network.

If there is no difference between the CFCP value of country A in the networks with and without country C, it means that country C is not affected by country A in the multi-step trade network, and if there is a difference in the CFCP value of country A, country C is being influenced by country A in the multi-step trade network. The sum of the difference between the CFCP values of countries A1, A2,…, An in the original network and the respective CFCP values when country C is removed is the degree of influence that country C experiences from other countries. This is called the vulnerable value. It reveals the extent to which country C is influenced by other countries in the network from a multi-step trade network perspective (Fig 3).

A high vulnerable value for a country implies that the removal of this country from the network results in significant changes in the CFCP values of other countries. This indicates that the country is strongly influenced by others within the multi-step trade network. On the other hand, a country with a low vulnerable value can be considered stable, as it experiences minimal fluctuations due to changes in other countries within the same multi-step trade network.

#### 2.3.2 Triggering value

**Fig 4:**
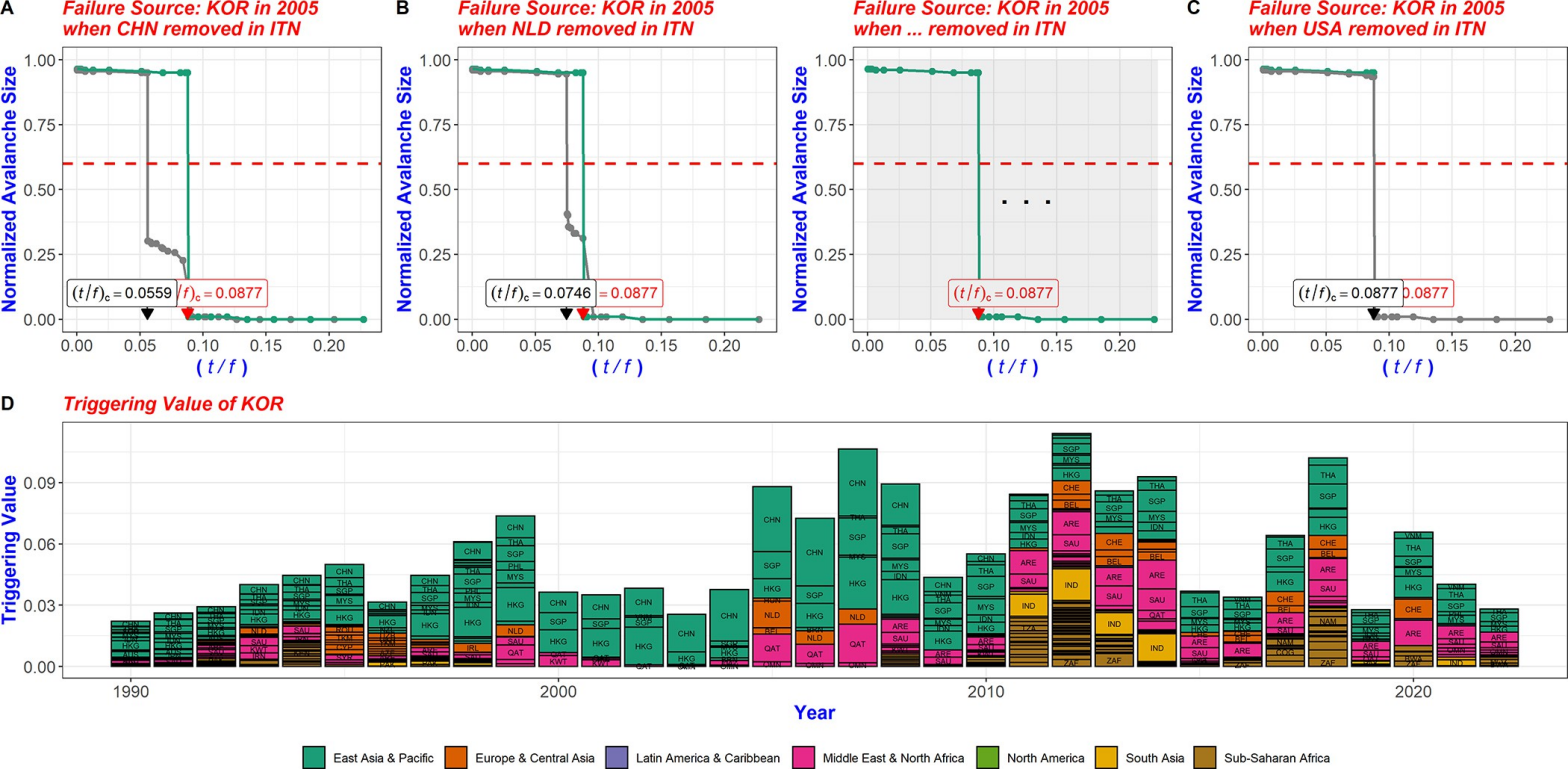
Triggering value and evolution of the node through the original CFCP of the failure-occurring country and the recalculated CFCP values when nodes are deleted. (A) Scenario when KOR is the epicenter of failure within the 2005 trade network: changes in CFCP value of KOR upon removal of CHN from the network. (B) Changes in the CFCP value of KOR upon removal of NLD from the network. (C) The CFCP value of KOR remains unchanged in the network both before and after the removal of USA. (D) Aggregated changes in the CFCP value of KOR comparing the original network and the scenario when each country is extracted from the network. This comparison illustrates the annual impact of KOR on other countries within the multi-step trade network. This is termed the triggering value.

The triggering value of a country, as depicted in Fig 4, is derived by comparing the CFCP value of that country (country A) in the original network with its CFCP values after removing other countries (C1, C2,…, Cn) from the network. The resulting values are summed to indicate how much influence the initial failure country (country A) has on other directly or indirectly connected countries (C1, C2,…, Cn). This triggering value quantifies the extent to which the country (country A) experiencing the initial failure affects other countries within the multi-step trade network.

A high triggering value indicates that if significant changes in the CFCP value of a country that experienced a failure occur when other nodes are removed from the network, then that country has a substantial impact on other countries within the multi-step trade network. On the other hand, a low triggering value implies that the country wherein a failure occurs has a limited impact on other countries within the multi-step trade network (See S2 Appendix for more detail).

## 3. Results and analysis

### 3.1 Evolution of cascading failure

CFCP is the critical point at which a failure in a country *i* reaches the load that will cause it to be transmitted to the world through the trade network. In a trade network that is directly or indirectly entangled, a failure that occurs in a country *i* affects only neighboring countries if its load does not exceed a critical point, but if the load exceeds a certain critical point, failure is transmitted to the connected countries all the way to the end of the trade network. Eventually, one country’s failure will be transmitted to most countries around the world. CFCP is a unique value for each country that is determined by the volume of trade with trading partners and GDP. If a country’s CFCP value is high, it means that a failure in that country can easily propagate across the world even with a small load, and that country has a large influence on the trade network.

**Fig 5:**
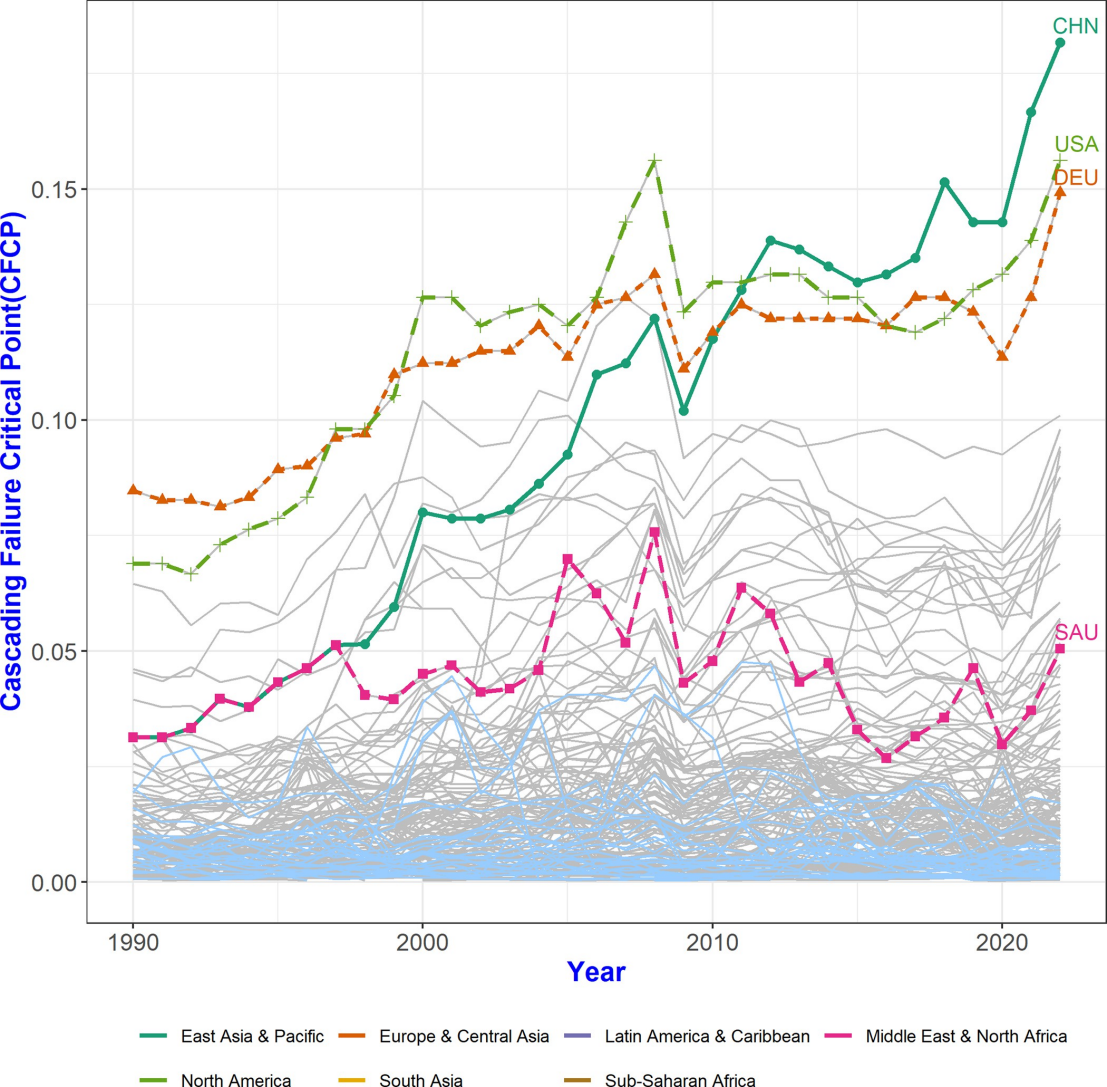
Evolution of CFCP values in each country. CFCP values have increased in most countries over the past 33 years. This means that even with a smaller failure load, failures can be propagated worldwide, and the load sensitivity to cascading failures has increased. There are 32 countries with lower CFCP values in 2022 than in 1990, most of which are countries with low GDP values (light blue); there are 173 countries with higher CFCP values in 2022 compared to 1990 (grey).

Fig 5 portrays changes in CFCP in the trade network from 1990 to 2022. The CFCP values in many countries trend upward over time, indicating that the sensitivity of each country’s failure load has increased. We interpret this as follows: the potential for a given failure to spread to the world is growing. Countries such as CHN, the USA, and DEU are at the top in terms of CFCP; these are countries whose failures can spread across the world at a lower load rate than those of other countries. In particular, the CFCP value of the USA decreased starting with the financial crisis in 2008 and reversed in CHN in 2011. This implies that although some fluctuations occurred after 2011, there is a high possibility of a cascading failure spreading across the world due to more sensitivity to the failure load of CHN than to the failure load of the USA.

In fact, China’s economy has grown rapidly since the 1990s, and multi-national companies around the world have expanded their local production networks centered on China. In 1990, CHN’s influence in the multi-step trade network was not so great, but in 2020, the country moved to a position where its failures can spread to the world even with a smaller load.

CFCP values for most high CFCP countries were higher in 2022 than in 1990. However, some countries had higher CFCP values in 1990 than in 2022. The CFCP values of GBR, AUS, and SAU either remained constant or decreased from the mid-2000s to 2022. In the case of SAU, the country’s CFCP value peaked in the mid-2000s and then continuously decreased. It is interesting that the influence of oil-producing countries did not increase compared to the influence of non-oil-producing countries. This may be explained by the fact that some regions may seek to reduce their dependence on a limited number of suppliers [20].

**Fig 6:**
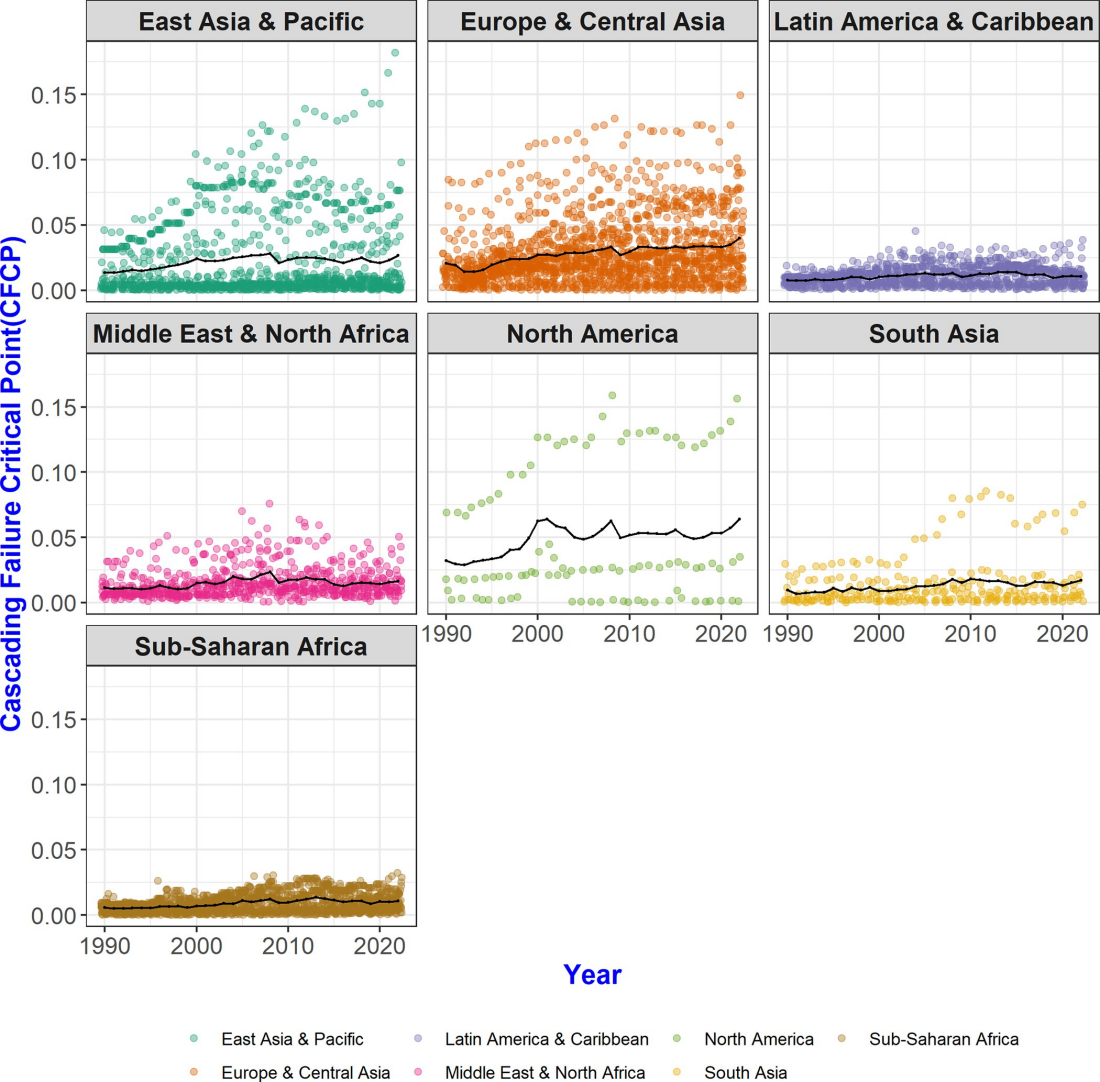
Regional distribution of CFCP values and mean. The distribution of CFCP values reveals distinct characteristics in each region. Notably, CFCP values vary widely in the East Asia & Pacific region and Europe & Central Asia region, whereas the distribution is more clustered with minimal standard deviation in the Latin America & Caribbean region and Sub-Saharan Africa region.

When examining the CFCP values of each country by region (Fig 6), it becomes evident that the distribution of CFCP varies across regions. For instance, within the East Asian & Pacific and European & Central Asian regions, the distribution of CFCP values is notably wide, whereas in the Latin American & Caribbean and Sub-Saharan African regions, the dispersion of CFCP values is relatively narrower, indicating a more concentrated distribution.

Similarities in the distribution of influence among some regions may occur in the multi-step trade network. In our study, we explore and confirm variations in interactions between these regions rather than individual countries within the network. Certain characteristics, which might not be evident when examining individual countries, may become apparent when we analyze countries grouped by region.

### 3.2 Evolution of vulnerable values and their influence on other countries

The vulnerable value of country A reflects the extent to which the CFCP values of other nodes in the network have increased because of country A and its connections. This value gives us an idea of the impact of other countries on country A within the context of the multi-step trade network. If its vulnerable value is high, it means country A is greatly impacted and quite dependent on the actions of other countries. If the value is low, then country A is less affected by and more independent from what others in the multi-step trade network do.

**Fig 7:**
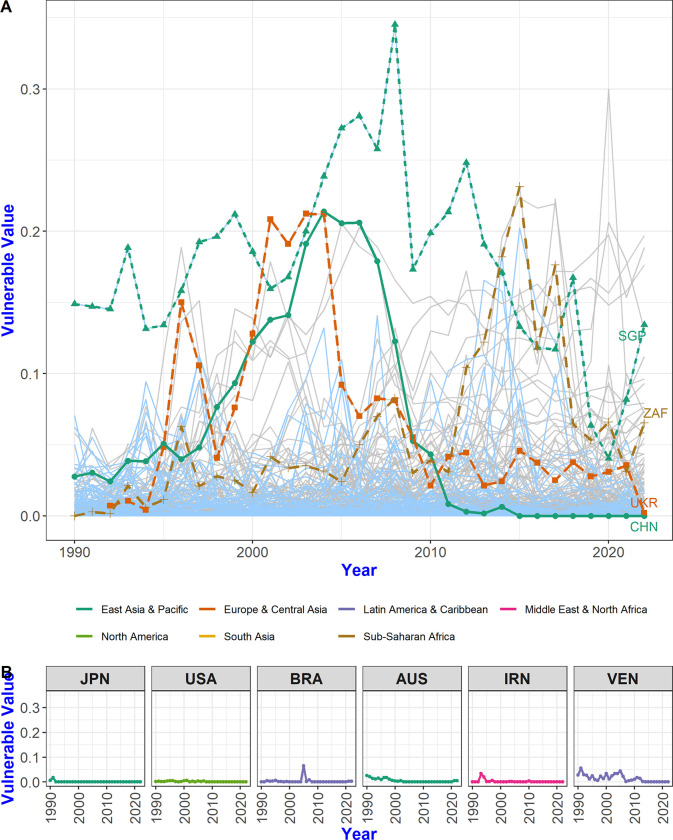
Evolution of vulnerable values of each country. (A) The vulnerable value indicates the degree to which a country is affected by other countries in terms of the possibility of cascading failure in the trade network. UKR, CHN, and SGP were most strongly influenced from 2001 to 2004, in 2004, and in 2008, respectively, but this influence gradually decreased as these countries developed independent trade ecosystems. In 2015, the influence of other countries on ZAF peaks and then declines again. (B) Some countries rich in natural resources or advanced economies have vulnerable values close to 0.

Fig 7 shows the extent to which each country has been affected in multi-step trade networks over the past 30 years. The vulnerable values of SGP (Singapore), UKR (Ukraine), CHN, and ZAF (South Africa) peaked between early 2000 and 2015 and then decreased until 2020. These trends are quite similar to those observed in GVCs throughout the world. Since the 1990s, China has taken on the role of the world’s factory by participating more actively in supply chains. Singapore increased its involvement in transit trade until the global financial crisis of 2008, and African countries have been actively involved in the GVC by boosting the trade volume of resources, particularly through trading oil with China from 2010 to 2015. However, after the global financial crisis of 2008–9, multi-national companies with production facilities around the world switched to reshoring or nearshoring back to their home countries in a bid for independence from the global economic network. The GVC expanded worldwide in the 1990s and 2000s, but this growth began to slow down after the global financial crisis of 2008 [21] along with the decline in global economic growth and investment and the diminished impetus for liberalization. Protectionism also spread around the world due to trade disputes between the USA and China. Fig 7 shows that trends in global trade moved away from the interconnected and interdependent state relations that existed prior to the global financial crisis.

Countries with vulnerable values close to 0 differ across periods, but countries like Japan (JPN) and the United States (USA), in addition to Brazil (BRA), Australia (AUS), Iran (IRN), and Venezuela (VEN), consistently have values close to 0. This observation is particularly interesting because it highlights that countries typically considered to be advanced nations rich in natural resources [21, 22] tend to have relatively small vulnerable values. This trend can be explained by examining how vulnerable values are calculated. Countries with low vulnerable values are often in the initial or final stages of the overall trade process. They might export raw materials or finished goods directly to their domestic markets. This is reminiscent of the concept of high forward participation in the GVC. Countries with high forward participation in the GVC can be categorized into two main types: resource-driven economies focusing on primary product exports due to abundant natural resources, and advanced economies characterized by industries with strong R&D focus and minimal dependence on intermediate goods [23]. As countries that originally relied on the export of raw materials transition to manufacturing utilizing these resources, their participation paradigm may shift towards low-level manufacturing, signifying an increase in backward participation [21]. Increased backward participation accords with increased influence from other countries within the multi-step trade network and an increase in vulnerable values. China’s strategy of moving towards producing technology-intensive intermediate goods for export, as opposed to simply assembling final goods, aligns with its goal of increasing added value in its manufacturing sector [24–26]. This strategic shift is reflected in Fig 7, where we observe an interesting pattern in CHN’s vulnerable value: it increased from 1990 until 2005, a period during which China may have been more susceptible to disruptions in its supply chain. However, from 2010 onwards, there was a noticeable decrease in CHN’s vulnerable value. This decline suggests that during the 30-year study period, China has been moving towards a less vulnerable position in the supply chain, enhancing resilience by reducing dependence on other countries for critical components and intermediate goods. This shift in vulnerable value towards 0 indicates that China has made progress in strengthening its supply chain and increasing its self-reliance in critical manufacturing processes. Thus, our results provide an interesting reflection on China’s evolving role in the global manufacturing landscape.

**Fig 8:**
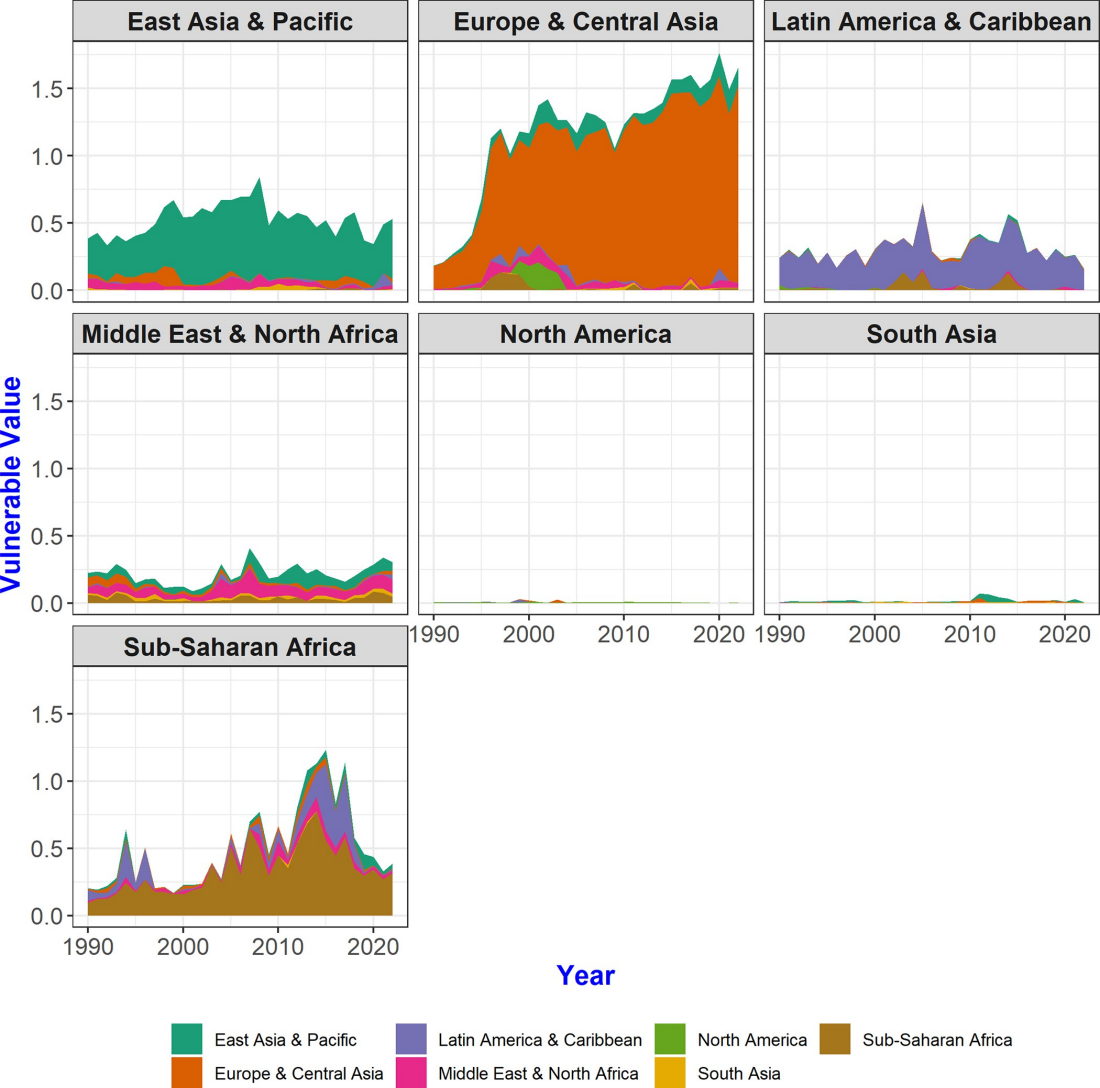
Evolution of vulnerable values by region. This figure illustrates the extent of influence originating from each region within the context of the multi-step trade network. The vulnerable values of countries within each region were aggregated. For instance, the first graph portrays the impact on the East Asia & Pacific region of other regions in the multi-step trade network. The chart reveals that countries within the East Asia & Pacific region wielded the most significant influence in the East Asia & Pacific region, while those in Europe & Central Asia and Middle East & North Africa regions exerted comparatively less influence.

Examining vulnerable values at the regional level offers a comprehensive view that captures the interdependencies and evolving patterns in the network, which might not be as apparent when examining individual countries. Economic integration, such as free trade agreements and customs unions, is often achieved in geographically close areas. By aggregating vulnerable values by region from the data on the impact between countries, it is possible to compare vulnerable values between regions. Fig 8 shows the evolution of the vulnerable values for each region. It reveals that most regions (except for the Middle East & North Africa, North America, and South Asia) have vulnerable values facilitating from the same region. In other words, the vulnerable value of the East Asia & Pacific region originates from the countries in the East Asia & Pacific region, which means that countries within the same region play a role in changing the value of CFCP for reasons related to regional economic integration.

The results of the analysis of CFCP value distributions across regions reveal similar patterns for the East Asian & Pacific and European & Central Asian regions, as well as for the Latin American & Caribbean and Sub-Saharan African regions (Fig 6). However, when observed through the lens of vulnerable values, additional insights emerge that were not apparent in the previous CFCP analysis. Though the East Asian & Pacific and European & Central Asian regions share the characteristic of geographical proximity to neighboring nations that exert substantial influence on them, vulnerable values for the East Asian & Pacific region were consistent in 2020 compared to 1990, reflecting an acquired stability. By contrast, over the past three decades, the European & Central Asian region experienced a notable increase in vulnerability, particularly due to the influence of countries within the same vicinity. When comparing Latin American & Caribbean and Sub-Saharan African regions, the Latin America & Caribbean region, representative of the Southern trading area, did not exhibit heightened vulnerability in 2022. For Sub-Saharan Africa, while vulnerability from intra-regional interactions continued to rise until 2015, the impact of the Latin American & Caribbean region became evident, as indicated by vulnerability spikes in the mid-1990s and mid-2010s.

### 3.3 Evolution of triggering values and their influence on other countries

From a country’s triggering value, it is possible to determine the impact of that country in an interconnected trade network. Countries with high triggering values have considerable influence on other countries in the multi-step trade network.

**Fig 9:**
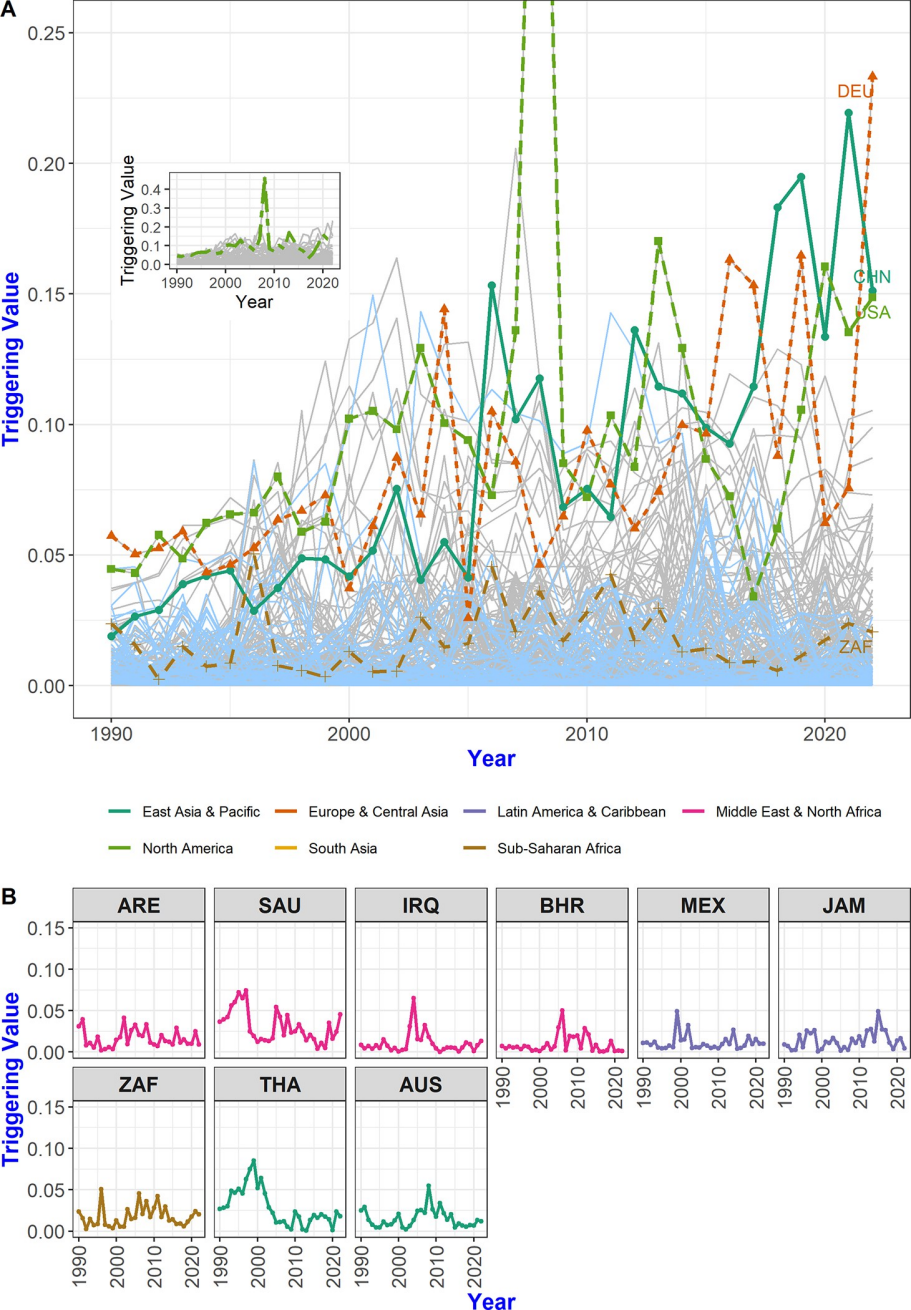
The evolution of triggering values of each country. (A) The triggering value indicates the degree of influence of a given country over other countries from a multi-step trade network perspective. During the global financial crisis of 2008, the triggering value of the USA soared; during this time, the USA had considerable influence on the possibility of cascading failure in other countries. The inset at the top left represents the entire range of the USA’s triggering value in 2008. (B) The observed decrease in triggering values for countries with primary resources, such as the UAE and SAU, suggests a diminished degree of influence on the sensitivity of other countries compared to the past.

In Fig 9, we see that the triggering value of the USA suddenly increased in 2008 and then decreased sharply again in 2009. The influence of CHN, DEU (Germany), and ZAF on other countries fluctuated in the middle years, but generally increased from 1990 to 2022. In the graph, the light blue line represents the 61 countries with lower triggering values in 2022 than in 1990, and gray represents 139 countries with the same or higher triggering values in 2022 than in 1990.

The year 2008 marked the beginning of the financial crisis in the USA. The substantial increase in the triggering value for this country suggests its significant influence over other nations. However, the USA’s impact on other network countries sharply diminished in 2009, coinciding with decreased trade and declining GDP following the 2008 crisis. As for CHN and DEU, their influence on other countries declined around 2005, fluctuating thereafter. Interestingly, periods of decline and resurgence are evident in the impact of these countries on other countries, with a notable increase in influence observed in 2022 compared to the early 1990s.

Among the 61 countries that experienced a decrease in their triggering values from 1990 to 2022, many have smaller economies. However, some countries also have large economies. This includes oil-exporting nations like the ARE (United Arab Emirates), SAU (Saudi Arabia), IRQ (Iraq), and BHR (Bahrain). Additionally, countries such as MEX (Mexico), JAM (Jamaica) from the Latin American & Caribbean region, ZAF from Sub-Saharan Africa, and THA (Thailand) and AUS (Australia) from the East Asian & Pacific region also fall into this category.

Countries that primarily concentrate on agricultural products and minerals, as opposed to high-tech manufacturing, are characterized by a decreased triggering value. This decrease signifies a reduced degree of influence on the sensitivity of other countries compared to the past. In the 2020s, amid a shift from the previous era of globalization expansion, countries predominantly reliant on primary resources are anticipated to have a minor ripple effect on the entire network.

**Fig 10:**
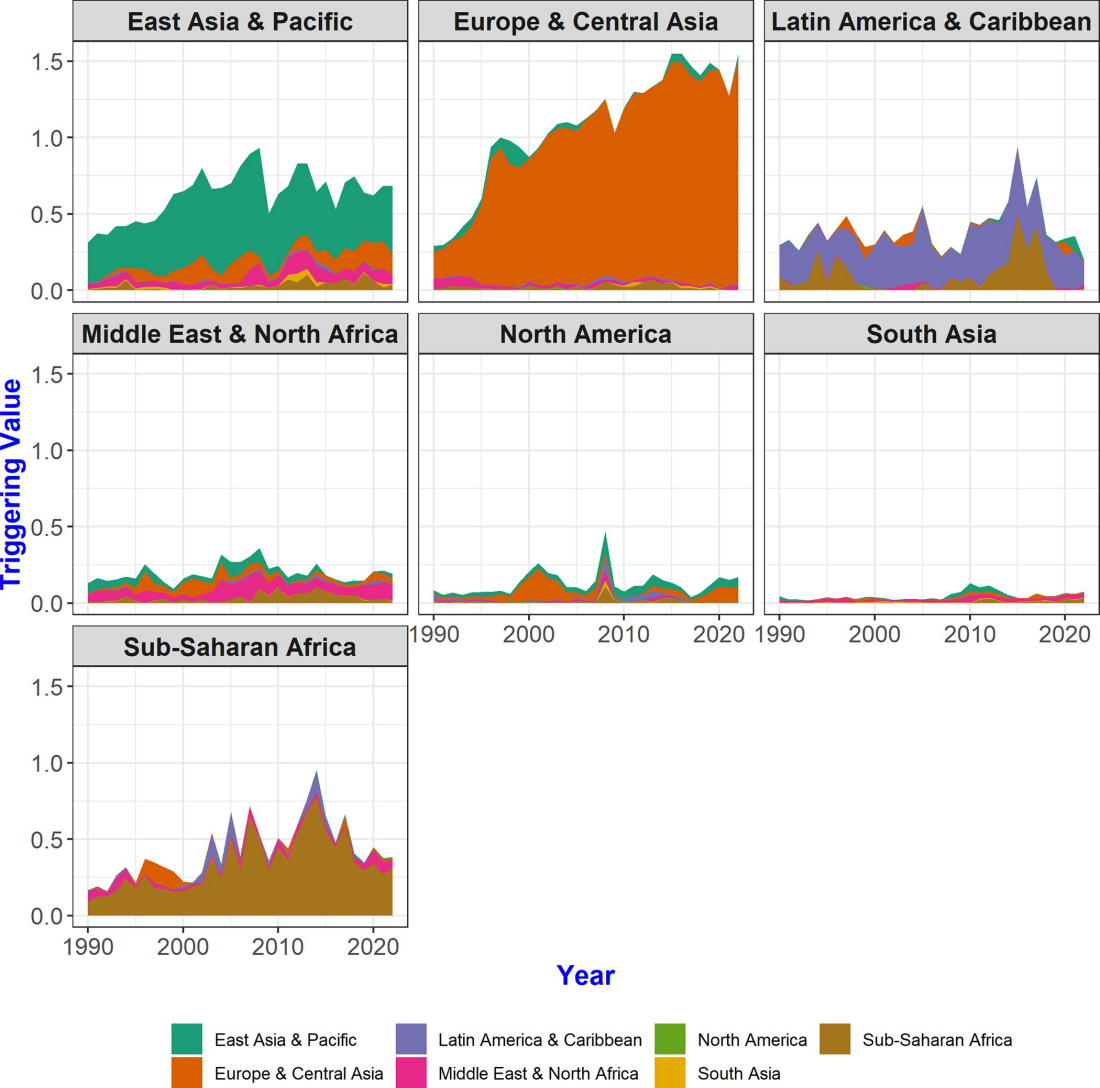
Evolution of triggering value by region. The aggregated triggering values of the countries belonging to each region. Influence of the countries in each region indicated by color. That East Asia & Pacific countries have the greatest influence on other countries in that region, followed by Europe & Central Asia, Middle East & North Africa, and Sub-Saharan Africa, while countries in the Europe & Central Asia region influence countries in that region at a much higher rate than other regions.

Examining the evolution of the triggering value by region reveals the influence of regions that affect other regions (Fig 10). Triggering values within the same region are pronounced across all regions except for North America and South Asia; comparison with these regions is difficult due to the small number of countries. Reasons include increased vertical specialization of production, integration of regional supply chains, and expanding intra-regional trade [27]. In particular, the increase in triggering values in the European & Central Asian region over the study period is worth attention. After the global financial crisis, the triggering values in this region, which had been increasing like in other regions, decreased, but they then increased again after 2009 and peaked in 2015. This confirms that countries in the European & Central Asian region had a great impact on other countries in the same region in terms of the multi-step trade network. This finding is consistent with the EU’s pursuit of economic integration through regional tariff elimination and common market policies.

Also unusual is that the Sub-Saharan African region accounts for a significant portion of the triggering values of the Latin American & Caribbean region in the 1990s and 2010s. This means that in the multi-step trade network, the Latin American & Caribbean region had a significant impact on Sub-Saharan Africa. During the 1990s, there was relatively limited trade between the Latin American & Caribbean and Sub-Saharan African regions in terms of exports and imports. Nevertheless, the results of this study confirm their significant influence on other regions, as indicated by the triggering values of the Latin American & Caribbean region. This result should be interpreted within the context of the multi-step trade network, where trade occurs across countries through multiple stages of import and export, starting with the resources.

In the 1990s, despite minimal direct trade, Sub-Saharan Africa and Latin America were indirectly connected through intermediary trade nations like the USA and European countries. These connections facilitated interactions. By the 2010s, trade partnerships had diversified and the multi-step trade network had expanded. Countries in the Sub-Saharan African and Latin American regions began actively engaging with a broad array of countries, including China, India, and Russia, in addition to the established trade centers of the USA, Europe, and Japan. This expansion played a role in the patterns of influence within the trade network as revealed by this simulation analysis.

Nowadays, trade between Latin America and Sub-Saharan Africa is carried out through third-party countries such as China, the USA, and EU countries. These trade links have provided mutual benefits and strategic trade opportunities for these two regions where economic links were traditionally weak [28]. Although intermediary trade by third-party countries has not been extensively researched in the past, it has been noted that third-party countries can play an important role in economic linkages between the two regions [1, 29]. This may be the cause of the significant impact of the Latin American & Caribbean region on the Sub-Saharan African region in the 1990s and 2010s.

## 4. Discussion

Traditional international trade has historically involved producing goods within a single country and exporting them as final products to foreign consumers. By contrast, contemporary trade is characterized by the movement of raw materials across multiple countries, ultimately converging in a final production location. This process often involves intermediate parts and components traversing multiple borders, contributing value throughout the entire supply chain.

**Fig 11:**
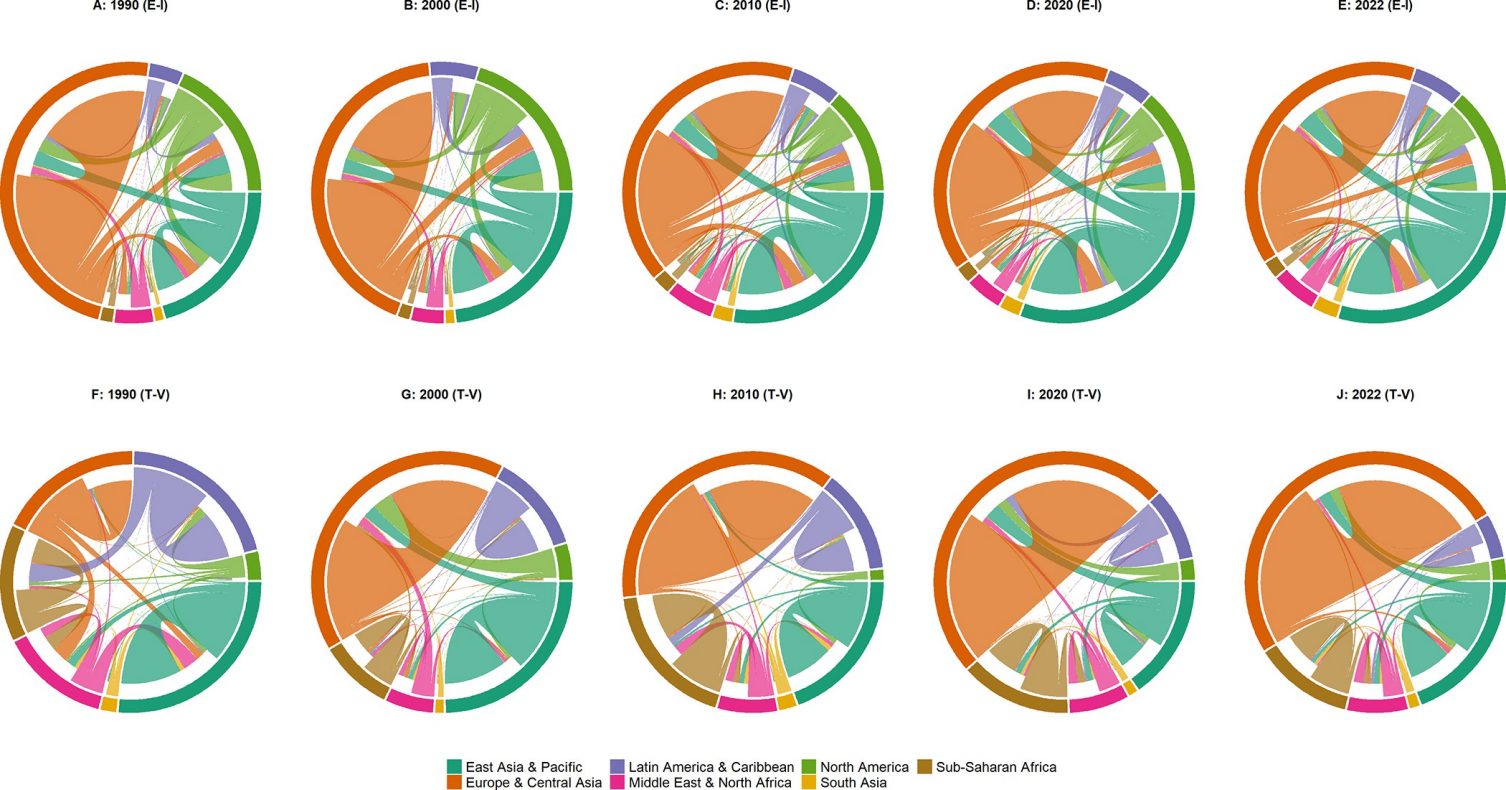
Comparison of export-import trade volume by region and triggering-vulnerable values by region. The world is divided into seven regions, each with its own color. (A)~(E) The outer ring represents the exporting region, and the inner ring represents imports. (F)~(J) For each of the seven distinct regions, triggering values are displayed on the chords connected to the outer ring, and vulnerable values are displayed on the chords connected to the inner ring. Vulnerable values indicate how much influence is received from a given region, and triggering values indicate how much influence is exerted on a given region. For example, the North American region has only an outer ring but no inner ring, showing that it is not affected by other regions, but that it does affect Europe & Central Asia and East Asia & Pacific regions.

Fig 11 illustrates the distinction between the relationships that can be observed through trade analysis and those revealed by triggering values and vulnerable values. The graphs denoted as (A) to (E) in the upper section of Fig 11 depict the volume of international trade using the export-import by region format. On the other hand, the diagrams labeled as (F) to (J) in the lower part of Fig 11 display triggering values and vulnerable values in a chord diagram layout. These diagrams showcase the usefulness of aggregated triggering values across regions, which consist of countries involved in failure propagation within the network. Additionally, they reveal the usefulness of aggregated vulnerable values across regions, which consist of countries that are affected by such failures.

Firstly, a consistent observation in the graphs depicting both the triggering values and vulnerable values is the substantial contribution of intra-regional trade to overall trade volume; in fact, it surpasses inter-regional trade. Similarly, a significant portion of influence in the multi-step trade network stems from intra-regional interactions, which impact the entire network considerably. This could be a natural result of geographic proximity and regional trade agreements [30, 31].

However, when observing regional trends in the proportion of the total from 1990 to 2020, distinct patterns emerge. In traditional terms, the European & Central Asian and East Asian & Pacific regions consistently engaged in a substantial amount of trade, maintaining a relatively stable pattern over the 30-year period. By contrast, the vulnerable values and triggering values for these regions tell a different story.

Interpreting the graph from the perspective of vulnerable values, we see that compared to the global export trade volume in 1990, the Middle Eastern & North African, Latin American & and Caribbean, and Sub-Saharan African regions had relatively high vulnerable values. A high vulnerable value means that a given country is highly affected by other countries in the same region. In 1990, countries that supplied energy resources such as coal and petroleum fuel were greatly affected by countries that demanded raw materials; thus, their regions were quite vulnerable. On the other hand, vulnerable values for Europe & Central Asia, which accounted for a significant portion of export trade volume in 1990, were low. However, the proportion of vulnerable values increased towards 2020, and the stability of this region decreased accordingly.

As for triggering values, countries in Europe & Central Asia in 1990 affected not only other countries within the region, but also those in other regions such as the Middle East & North Africa and East Asia & Pacific. By 2022, only the intra-regional effects of these countries are evident; they had no influence on other regions. This can be interpreted as follows: exports and imports from the European & Central Asian regions may originate in the East Asian & Pacific and North American regions, but are strongly affected by regional influences in terms of mutual exchange of raw materials and intermediate goods in the multi-step trade network. The vulnerable values and triggering values for these regions confirm the increased connectivity of countries in Europe & Central Asia; note that this could not be confirmed directly using trade data alone. In addition, these values confirm the degree of mutual influence between countries in the multi-step trade network.

There is also a relationship between the Sub-Saharan African and Latin American & Caribbean regions, which is not easily confirmed by data related to trade volume alone. The Sub-Saharan African region was significantly affected by the Latin American & Caribbean region in the 1990s and 2010s. As described previously, even between countries with no direct trade relationship, large vulnerable values may indicate that raw material trade occurs through intermediary countries.

Both the Sub-Saharan African and Latin American & Caribbean regions primarily export agricultural products like coffee, beans, fruit, cocoa, and tea, along with natural resources such as oil, gas, iron ore, copper, and gold [32]. Key trading nations often act as gateways for exports to other regions, serve as intermediaries (e.g., the Netherlands), or are major consumers of resources (e.g., the USA and China) [33]. While direct trade volume between these two regions might not be substantial compared to trade between other regions, the graph depicting triggering values and vulnerable values provides insights into their interactions. This graph reveals instances where the Latin American & Caribbean region impacts the Sub-Saharan African region (1990, 2010) and vice versa (2005). Presumably, when a failure occurs in a pivotal trading country within either Sub-Saharan Africa or Latin America, or in a connected country in another region, propagation of that failure is amplified through these key countries at multiple trading stages. Ultimately, this triggers a change in the vulnerability of countries in other regions, even those not directly involved in the trade.

For instance, the simulation results indicate that an export disruption in cotton, a major commodity, due to a failure in BEN (Benin) in Sub-Saharan Africa in 2005 affected the vulnerability of PAN (Panama) and TTO (Trinidad and Tobago) in the Latin American & Caribbean region. This unique insight emerges from this study’s findings and was not evident in previous research in this area based on traditional trade volume data.

The regional data on triggering values and vulnerable values highlights another interesting fact–the influence between regions is not always reciprocal. In other words, if region A significantly impacts region B, it does not necessarily mean that region B will have a similarly significant influence on region A. For instance, looking at the data for 1990, we see that Europe & Central Asia had an influence on the Middle Eastern & North African region, while the Middle Eastern & North African region influenced the East Asian & Pacific region. Similarly, the Latin American & Caribbean region affected the Sub-Saharan African region. In this latter case, nearly 50% of the vulnerable values generated in the Sub-Saharan African region can be attributed to failures in the Latin American & Caribbean region. Now, comparing this to the situation in 2022, we see that the European & Central Asian region is influenced by other regions, but its impact on other regions is relatively insignificant. This suggests that, compared to 1990, in 2022, failures are more likely to be propagated to countries within the European & Central Asian region when they occur in another country within the same region. This clearly illustrates the evolving dynamics of regional trade interactions and the cascading effects of failure in the multi-step trade network.

Additionally, though the considered period may not be sufficient to establish definitive trends, our analysis results shed light on how the COVID-19 pandemic has impacted global trade networks from 2020, when the full effects of the pandemic were first observed, to the most recent data in 2022. Numerous scholars have deliberated on the pandemic’s impact on supply chains, highlighting that disruptions in the supply chain can propagate from raw material procurement to the consumption of final products, potentially resulting in a large-scale global crisis [34]. The findings of this study reveal a general decrease in the CFCP value for most countries in 2022 compared to 2019. This decline can be attributed to extensive lockdown policies implemented by most countries and an overall reduction in trade volume. However, the magnitude of this decline is not as pronounced as during the 2008–9 financial crisis, and the period is not long, so there is an observable upward trend post-2021 (Fig 5). This indicates that, although sensitivity to changes in trade volume leading to global failure temporarily decreased due to reduced mutual trade volume, the long-term trend in load sensitivity remained the same. In other words, the trend toward increasing failure transmission between countries continued after the occurrence of COVID-19 as was seen before the COVID-19 pandemic [35, 36]. Vulnerable value data from 2020 to 2022 does not deviate from the general trend observed from 1990 to 2020. Over time, the vulnerable values of many countries decreased, revealing them to be gradually less vulnerable to the influence of other countries. However, over this same relatively short period (between 2020 and 2022), there is a slight increase in influence from other countries in the same region, particularly in the East Asia & Pacific and Europe & Central Asia regions (Fig 8). This mirrors the previously mentioned overall trend in the supply chain, which is shifting from globalization to regionalization before the COVID-19 pandemic [35–38]. Many multi-national companies that previously relied on China as a supplier of intermediate parts began diversifying their supply chains in nearby countries due to the US-China trade dispute starting in 2018 [36, 39]. This shift may have contributed to an expansion of mutual influence and changes in load sensitivity between countries within the East Asia & Pacific and Europe & Central Asia regions, respectively.

## 5. Conclusion

We herein presented a quantified index capable of measuring how production or supply failures in one country affect countries around the world directly or indirectly due to their position in connected trade networks. We also explored the evolution of each country’s influence in the multi-step trade network from a macroscopic perspective.

Overall, our index developed for this study provides valuable insights into the evolving dynamics of multi-step trade networks. In addition, our results are similar to those for the GVC, highlighting the changing patterns of influence among countries and regions.

Summarizing the aforementioned results, we now draw the following conclusions.

Firstly, sensitivity to changes in trade volume, where a country’s failure is sequentially transmitted to directly and indirectly connected countries worldwide within the trade network, has increased in many countries such as CHN, USA, and DEU. In other words, when a failure occurs in one country, that failure spreads globally, as indicated by even relatively small changes in load. By contrast, a different trend may be observed regarding the influence of oil-producing countries, which previously held significant sway over related industries. This shift is a result of the changing role of oil-producing countries within the supply chain. The demand for clean fuels is on the rise due to concerns surrounding climate change; for example, technological advancements have made shale gas production a viable alternative to crude oil in the USA. Consequently, the price of crude oil has been affected. In response to these developments, oil-producing countries are actively working to diversify their national income, which was historically heavily reliant on oil revenues.

Secondly, despite the increased sensitivity to changes in trade volume globally, many countries are experiencing a reduced impact from other countries within their multi-step trade networks. In fact, in the multi-step trade network, countries such as JPN, USA, ITA (Italy), and FRA (France) have already reached a stage where they are almost unaffected by other countries. For CHN, there was an initial increase in influence from other countries during the study period, particularly from 1990 to 2005, as the country began to acquire manufacturing plants all over the world. However, this influence gradually diminished over time, and since 2015, the degree of influence from other countries on China has become almost negligible. It appears that various countries have taken steps to establish independent supply chains in order to mitigate vulnerabilities caused by external factors.

The third key insight derived from our study involves vulnerabilities caused by other countries within the trade network from a regional perspective. This analysis revealed a noteworthy aspect that was not evident in the simple trade network analysis. Despite relatively lower levels of direct trade connectivity, substantial interaction occurred between the Latin American & Caribbean and Sub-Saharan African regions. This suggests that these regions are linked through intermediary countries within a hidden multi-step trade network. Through this network, countries in the Latin American & Caribbean region may influence load sensitivity and, consequently, global failure patterns in Sub-Saharan Africa. To put it simply, any abrupt cessation of trade by a Latin American & Caribbean country due to factors like natural disasters or economic crises could alter the load sensitivity to global failure in Sub-Saharan African countries, consequently impacting their vulnerability.

Furthermore, most regions exhibited mutual influence among countries within the same region. The results of the analysis revealed a weakening trend in intra-regional interactions in the East Asian & Pacific region, whereas they indicated a gradual increase in such interactions in Europe & Central Asia. Many studies have discussed the growing significance of regional influence, attributing it to factors like geographical proximity and the proliferation of trade agreements and customs unions aimed at fostering economic integration within regions. In terms of the sensitivity of load-induced global failure, our findings revealed a substantial surge in vulnerability among countries within the European & Central Asian region, particularly when compared to other regions. This underscores an increase in the vulnerability of countries interlinked with Europe & Central Asia to the cascading repercussions of failure within this region.

The COVID-19 pandemic had a temporary reductive impact on connectivity in 2020, but in general, no significant deviation from ongoing trends [35] was evident. Strict lockdown policies implemented by countries created temporary difficulties in sourcing intermediate goods within supply chains, reducing sensitivity to changes in trade volumes that could trigger global failures. However, signs of recovery started to emerge in 2021. Additionally, there was a noticeable trend toward resilience in multi-step trade networks as many countries became less vulnerable to the influence of other countries (Fig 7). Nevertheless, intra-regional influence gradually increased, reflecting the broad trend of deglobalization and regionalization that was already underway before the COVID-19 pandemic.

The argument stating that stronger trade does not necessarily indicate a higher risk of crisis transmission [40] is relevant to the findings of this study. Researchers have argued that trade connections facilitate the transmission of shocks across countries as a whole [41, 42], and that excessive trade links can potentially have negative consequences [43]. The results of this study provide insights into how certain countries, despite not being major players in the overall trade network, still have significant influence in the global supply chain.

Agent-based models are valuable for understanding how network structures can affect macroscopic outcomes. However, as with many studies utilizing agent-based models, this study has certain limitations; for example, the results do not elucidate how these networks were originally formed or how they might evolve in the future [44]. Another limitation is connected to the assumptions on which this simulation is based. There is a gap between the simulation and reality in terms of a given country’s trade statistics, its capacity to resist crisis, and the size of the transmitted crisis. There are also limitations with the data. Although the share of exported services has gradually increased, this increase is difficult to reflect accurately in overall export statistics if services are exported indirectly in the form of intermediate inputs. Also, the IMF data does not include import and export data for Taiwan. Therefore, a significant portion of the multi-step trade network is excluded from the analysis in this study. However, with respect to the role of each country in the trade network, our results are meaningful in that we pay attention to the overall composition of the network and the interactions within it. This is why we obtain results that accurately reflect the situation in the supply chain from a holistic point of view. The international division of labor in many economic activities, from production to sales, requires a new analytical approach to world trade flows on a global scale. Our study provides that approach.

Close interconnections between countries in trade networks can facilitate the transmission of shocks or failures from one country to another. Unexpected occurrences such as the pandemic may further accelerate this trend. Better understanding of how failures spread from one country to another can mitigate intentional or unintentional disconnections caused by pandemics, large-scale natural disasters, or political strife and aid in anticipating future global trade developments. Now is an opportune moment to apply insights such as those offered in this study for the development of effective response strategies. The vulnerable value and triggering value measures presented herein can be utilized to identify key nodes within the supply value chain that are particularly susceptible to change or disruption. By identifying the vulnerabilities of these specific nodes, policymakers and analysts can formulate strategies to address potential risks, enhance resilience, and maintain network stability. A proactive approach will enable them to manage and navigate the evolving dynamics of trade networks effectively, ensuring the stability and continuity of global supply chains.

## Supporting information

S1 Data Node data: GDP in current USD(millions) from 1990 to 2022. (ZIP)

S2 Data Link data: Export and import in current USD(millions) from 1990 to 2022. (ZIP)

S1 Appendix Process of calculating (*t*/*f*) and CFCP. This document contains the detailed process on how to determine the appropriate (*t*/*f*) value.
(DOCX)

S2 Appendix Supplementary explanation of Figs 1 to 4. In the manuscript, 2005 data was used as it demonstrated the most significant changes. The methodology used for presenting the latest year’s data, 2022, remains consistent with that employed for the 2005 results.
(DOCX)
