# Supplementary material for: The potential for cascading failures in the international trade network
Source: PLoS One. 2024 Mar 1;19(3):e0299833. doi: 10.1371/journal.pone.0299833 (PMC10906889; doi:10.1371/journal.pone.0299833)
Supplement: S1 Appendix — This document contains the detailed process on how to determine the appropriate (t/f) value. (DOCX) [file pone.0299833.s003.docx]

## S1 Appendix. Process of calculating $\mathbf{(}\boldsymbol{t}\boldsymbol{/}\boldsymbol{f)}$ and CFCP

This simulation is based on the following process: The network is represented as a weighted directional network, where nodes represent countries based on their respective Gross Domestic Products (GDPs), and edges denote the magnitudes of import and export flows between these countries. Each node is characterized by its capacity, denoted as $C_{i}$, and each link possesses a weight, denoted as $W_{ij}$.

Let us consider a scenario where a particular country, represented by node $i$, undergoes an initial failure. Following this failure, the weights ($W$) of all links associated with country $i$ are reduced by a fraction, denoted as $f$. If the total reduction, $\Delta W$, in either the incoming or outgoing link weights of any countries connected to country $i$ exceeds a fraction, $t$, of its node capacity ($C$), then these connected countries also collapse and experience a failure. The link weights of the failed countries decrease by the fraction $f$, triggering a cascade of failures if the accumulated reduction, $\Delta W$, exceeds that country’s fraction, $t$, of its node capacity ($C$). The process continues until there are no more failed countries in the network, and the number of failed countries in the network is referred to as Avalanche Size.

The method employed for calculating Avalanche Size in this study shares the same process as the model outlined in the previous study [22]. However, notable distinctions exist between the two studies.

In the model presented in the previous study, a specific ratio $(f/t)$ exhibiting a power law distribution in the Avalanche Size distribution was identified. This $(f/t)$ value, consistent across all countries, was then applied to determine the Avalanche Size when failure initially occurred in each country. This allowed for a comparison of the Avalanche Size of each country under the same failure load ($f$), facilitating the evaluation of each country's influence.

In contrast, this study expands the approach by considering all $(f/t)$ ratios of each country. Here, $(t/ f)$ is used instead of $(f/t)$ to intuitively interpret the size of the influence of each country, as shown later. When a failure occurs in one country, each Avalanche Size is computed based on the change in the $(t/ f)$ value. This study shows that in every country, at any given $(t/ f)$ ratio, avalanche size is not limited to one region, but is found to be rapidly expanding worldwide. (S1 Fig) This specific $(t/ f)$ value is defined as the Cascading Failure Critical Point (CFCP), and this process is systematically applied to all countries to obtain all countries’ CFCP values. This methodology, focusing on the CFCP of each country, provides valuable insights into the potential for cascading failures and the evolving dynamics of the multi-step trade network. It offers a detailed understanding of how failures propagate globally, highlighting each country's influence on the network's most peripheral countries.

**
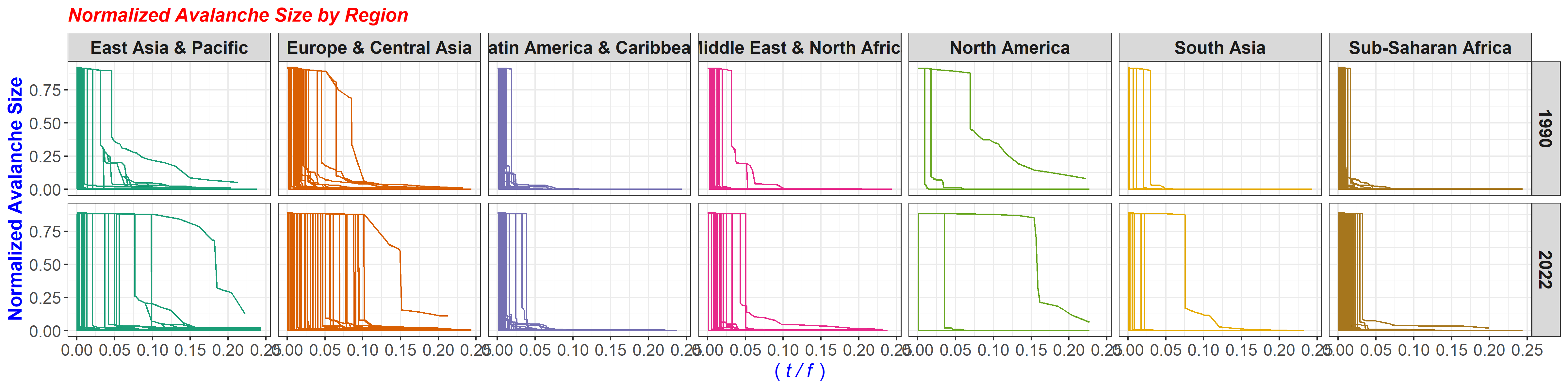
**

**S1 Fig. Normalized avalanche size according to changes in** $\mathbf{(}\boldsymbol{t}\boldsymbol{/}\boldsymbol{f)}$

A distinctive stepped pattern emerges when plotting the avalanche size for all countries while varying the $(t/ f)$ value. This signifies that all countries, to varying extents, have the potential to propagate failure globally at specific $(t/ f)$ levels. For better readability, the figure is segmented by region.
