# Supplementary material for: The potential for cascading failures in the international trade network
Source: PLoS One. 2024 Mar 1;19(3):e0299833. doi: 10.1371/journal.pone.0299833 (PMC10906889; doi:10.1371/journal.pone.0299833)
Supplement: S2 Appendix — In the manuscript, 2005 data was used as it demonstrated the most significant changes. The methodology used for presenting the latest year’s data, 2022, remains consistent with that employed for the 2005 results. (DOCX) [file pone.0299833.s004.docx]

## S2 Appendix. Supplementary explanation of Figs 1 to 4

Even for the graph generated for the latest year, 2022, the methodology employed remains consistent with that used for the 2005 results presented in the manuscript. (As part of the revised version of the manuscript to assess the impact of COVID-19, data for two additional years, 2021 and 2020, were included.) However, considering that the most significant change is observed in the year 2005, it is included as an illustrative example in the manuscript to enhance clarity. The graphical outcomes for 2022, reflecting the most recent data, are as follows.

In the graph illustrating the $\boldsymbol{(}t/ f\boldsymbol{)}$ of KOR in 2022(S2A Fig), similar to the pattern observed in 2005, there is a noticeable surge in Avalanche Size, exhibiting a staircase-like upward trend at $\left( t/ f \right)_{c}$.


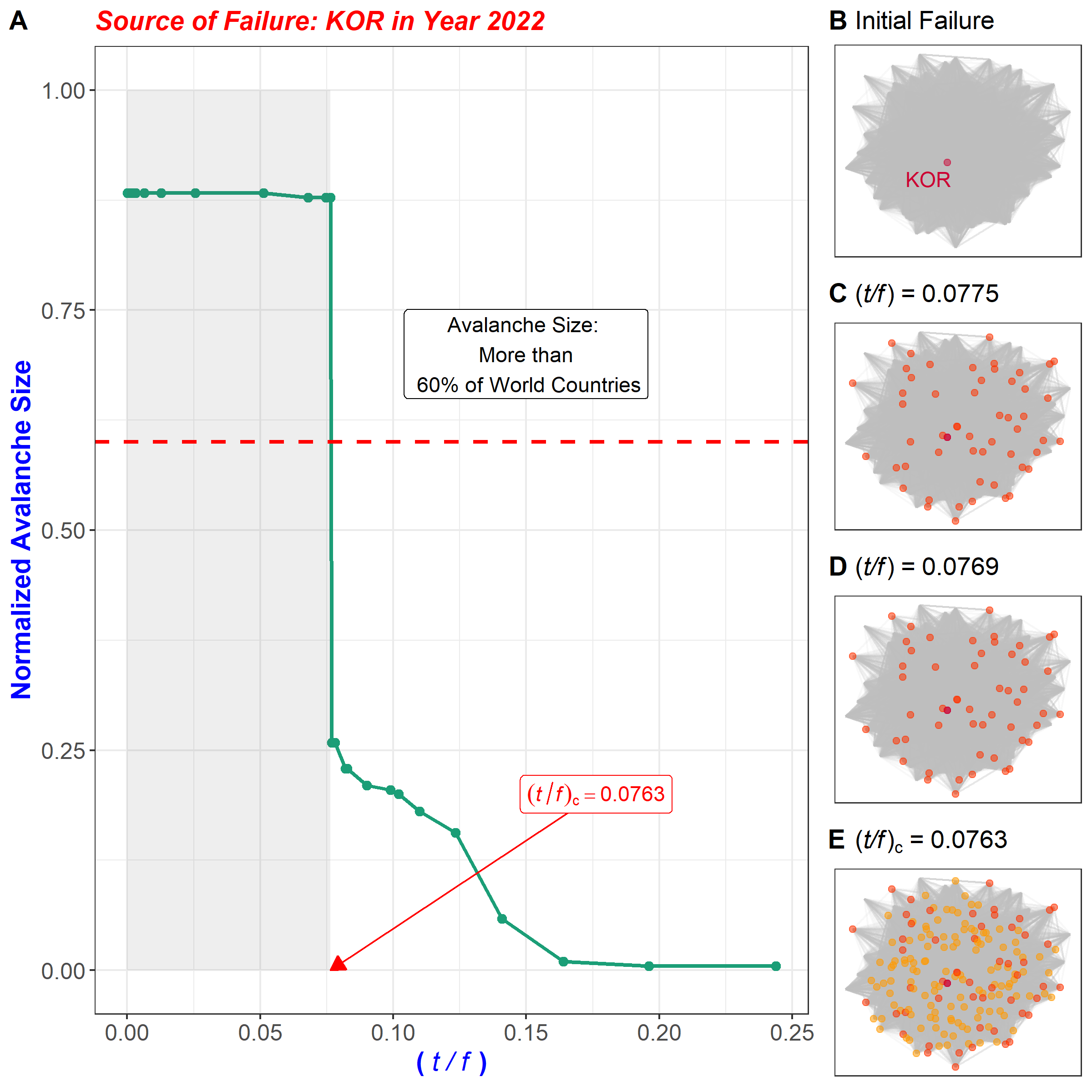


**S2A Fig. Normalized avalanche size with respect to changes in** $\boldsymbol{(t}\boldsymbol{/}\boldsymbol{f)}$ **value**

If CHN is removed from the 2022 trade data, there is no change in KOR's CFCP value(S2B Fig). This is because the original failure load required for the failure occurring in KOR in 2022 to propagate globally remains unchanged from the load needed for the failure to propagate worldwide in the network where CHN was removed. CHN is, therefore, not affected by KOR in the multi-step trade network of 2022.


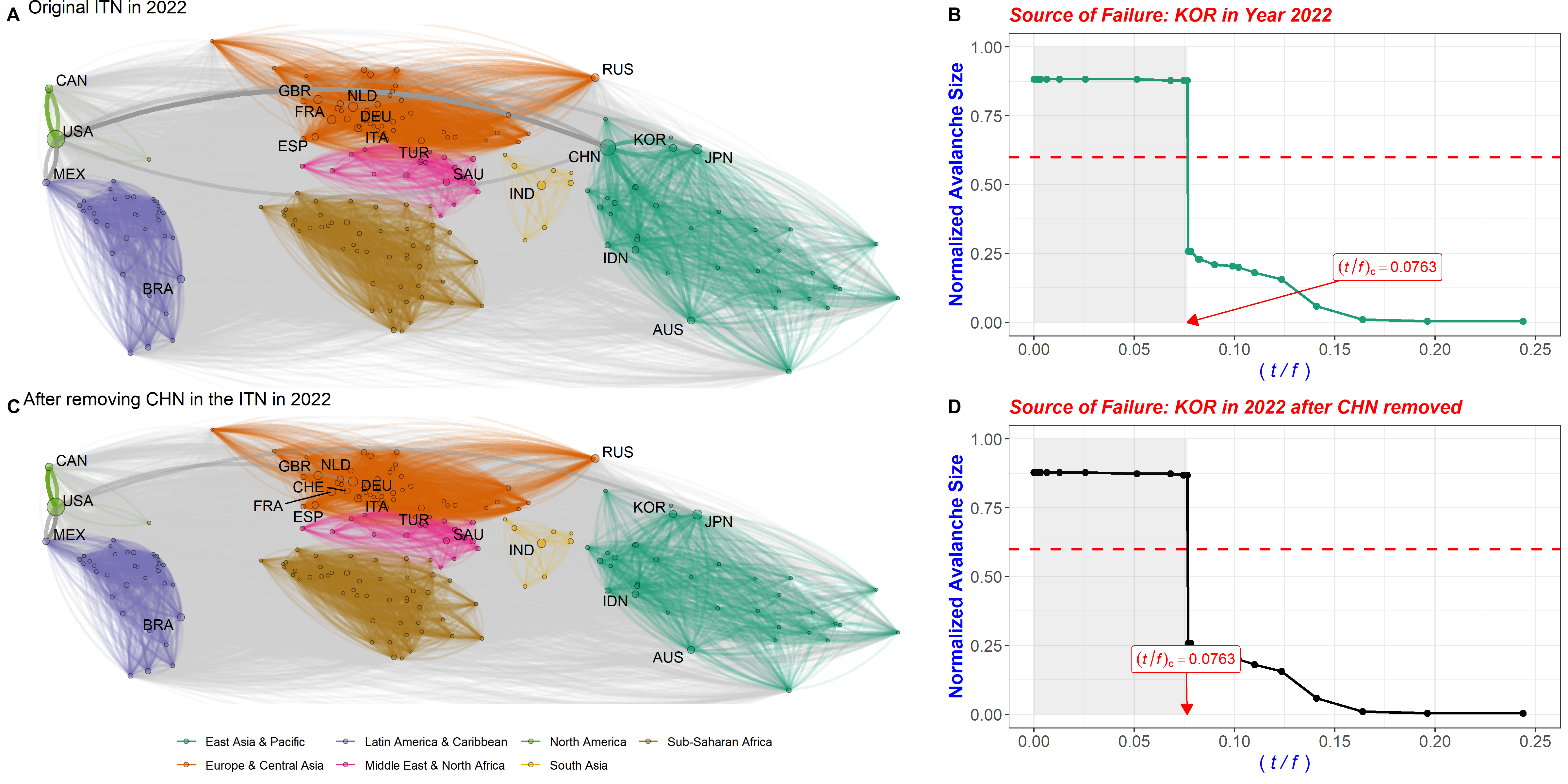


**S2B Fig. Changes in CFCP between the original trade network and the network after a specific node is removed**

Examining the trend of CHN's vulnerable value, the highest value in 2004 is noteworthy. In 2005, the year used as an example in the manuscript, it was influenced by THA, SGP, MYS, KOR, IDN, HKG, JPN, and USA(S2C Fig). However, this influence gradually decreased after 2006, reaching zero and remaining there from 2015 onwards. Thus, since 2015, there has been no influence from other countries on CHN. This result is consistently evident when CHN is removed from the 2022 trade network, showing no change in the CFCP for KOR.


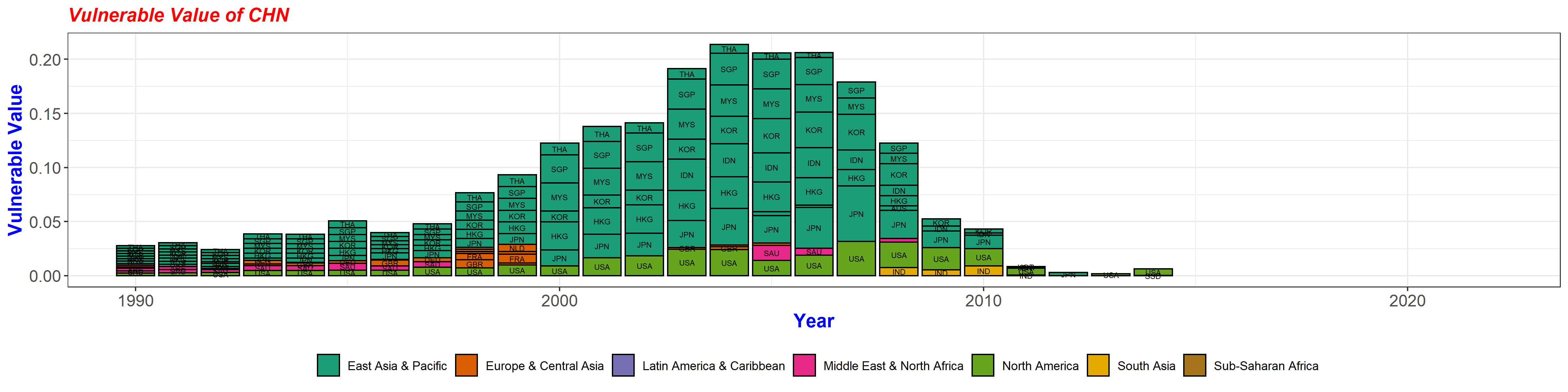


**S2C Fig. Vulnerable value of China (CHN) over time**

In the previous example, we observed changes in KOR's CFCP value when removing SGP instead of CHN from the original network (S2D Fig). Since SGP is influenced by KOR, removing SGP from the original network resulted in a change in the CFCP value of KOR.


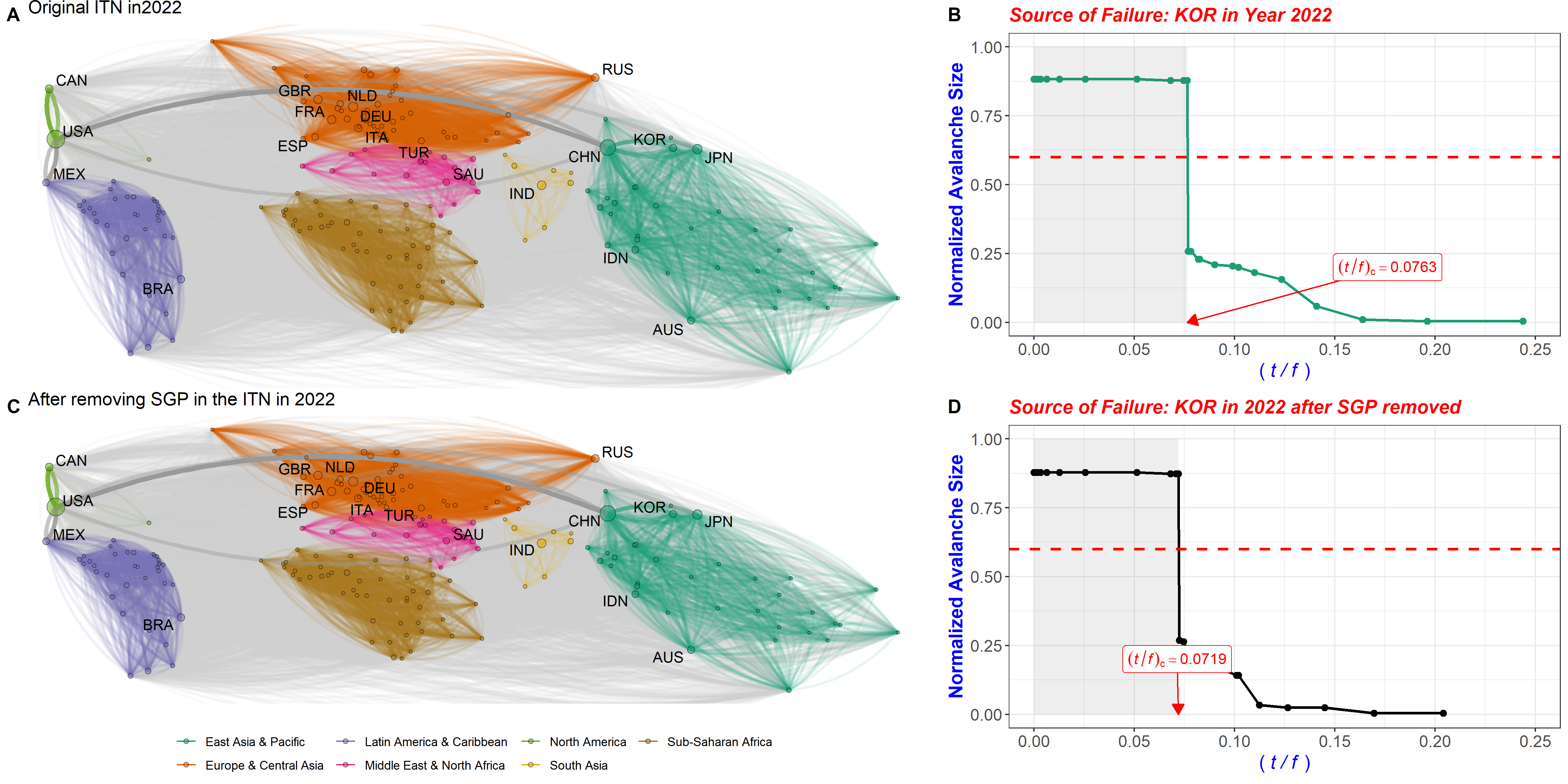


**S2D Fig. Changes in CFCP between the original trade network and the network after a specific node is removed**

When KOR is removed from the network in 2022, the CFCP values of SGP, USA, CHN, etc., undergo changes, but the CFCP of ARE does not change (S2E Fig). This is explained by the fact that KOR was influenced by SGP, USA, CHN, etc., but not by ARE. Countries contributing to the vulnerable value of KOR in 2022 include CHN, SGP, MYS, HKG, JPN, DEU, and USA, thereby indicating that in 2022, KOR was influenced by these specific countries.


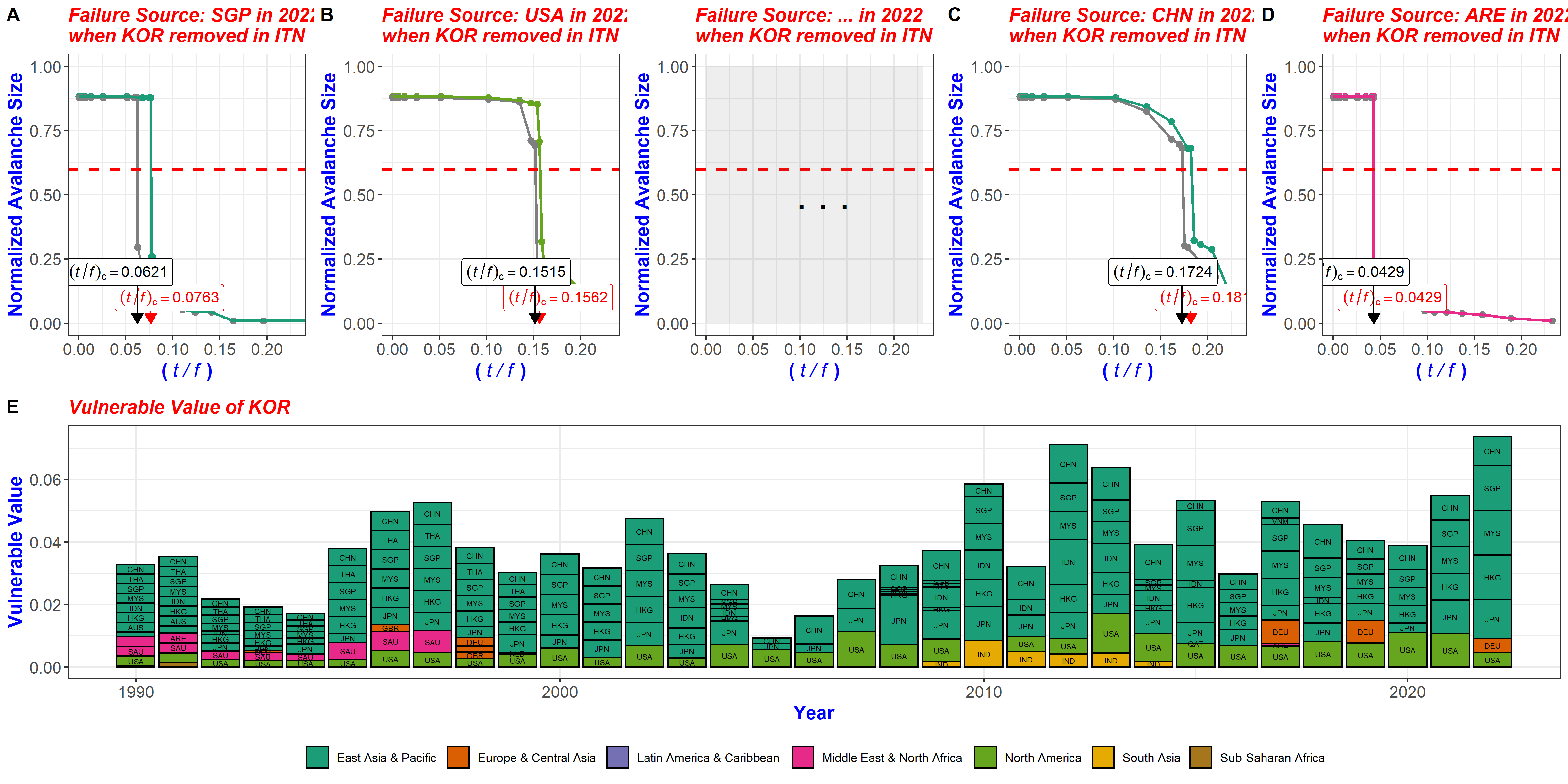


**S2E Fig. Vulnerable value and evolution of the node through node deletion and CFCP recalculation**

To comprehend the extent of KOR's influence in the trade network, we examined variations in KOR's CFCP when other countries are excluded (S2F Fig). In 2022, if SGP or ARE is removed, KOR's CFCP undergoes a change, whereas when USA or CHN is excluded, KOR's CFCP remains unchanged. Consequently, it can be inferred that KOR influences SGP or ARE, but does not have a discernible effect on the USA or CHN.


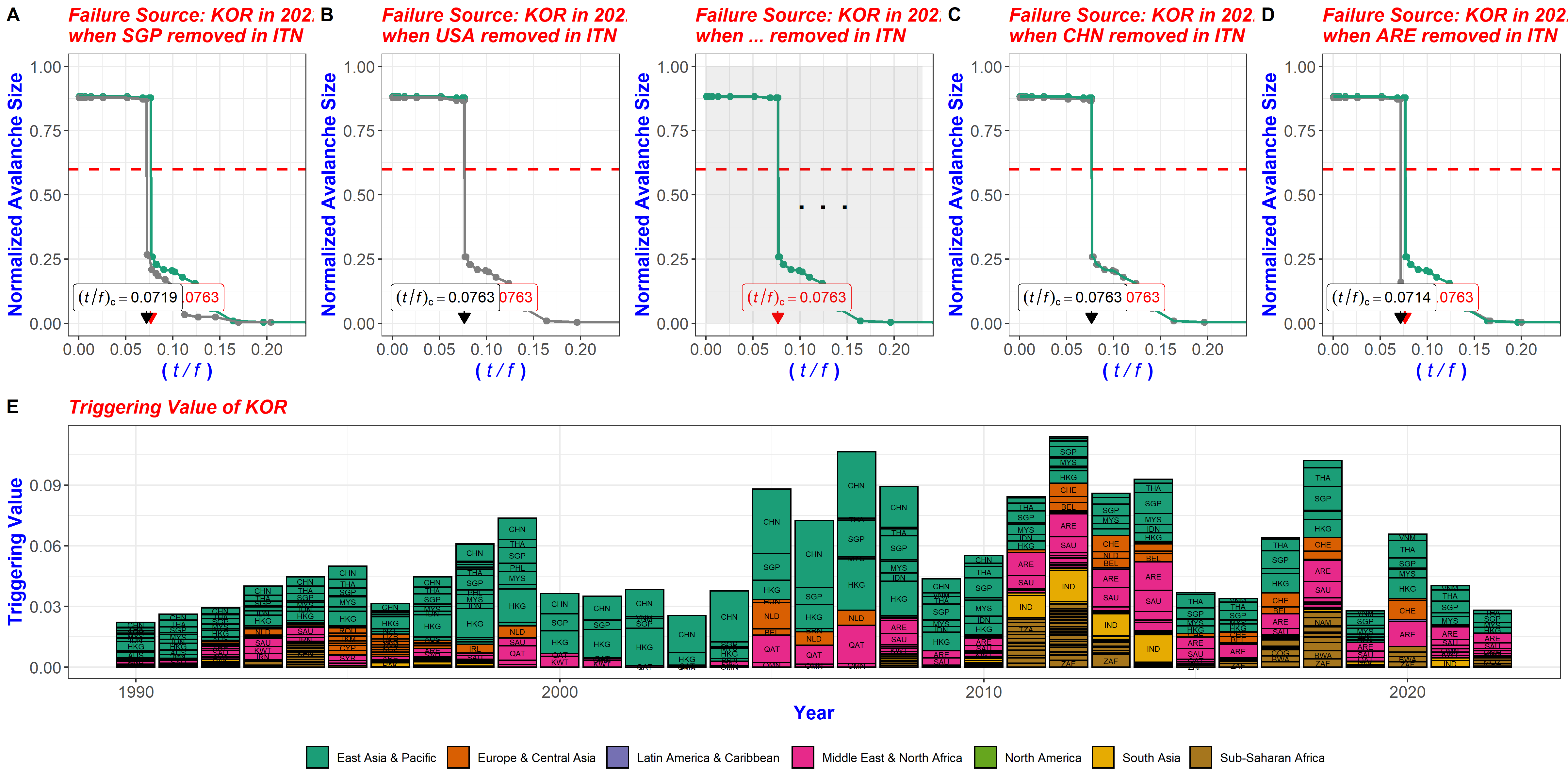


**S2F Fig. Triggering value and evolution of the node through the original CFCP of the failure-occurring country and recalculated CFCP values when nodes are deleted.**
